# Sustainable all-weather CO_2_ utilization by mimicking natural photosynthesis in a single material

**DOI:** 10.1093/nsr/nwad275

**Published:** 2023-10-28

**Authors:** Xianjin Shi, Yu Huang, Ran Long, Zhenyu Wang, Liqin Wang, Junji Cao, Gangqiang Zhu, Yujie Xiong

**Affiliations:** State Key Laboratory of Loess and Quaternary Geology (SKLLQG), Key Laboratory of Aerosol Chemistry and Physics, Institute of Earth Environment, Chinese Academy of Sciences, Xi’an 710061, China; Center of Excellence in Quaternary Science and Global Change, Chinese Academy of Sciences, Xi’an 710061, China; University of Chinese Academy of Sciences, Beijing 100049, China; State Key Laboratory of Loess and Quaternary Geology (SKLLQG), Key Laboratory of Aerosol Chemistry and Physics, Institute of Earth Environment, Chinese Academy of Sciences, Xi’an 710061, China; Center of Excellence in Quaternary Science and Global Change, Chinese Academy of Sciences, Xi’an 710061, China; Hefei National Research Center for Physical Sciences at the Microscale, School of Chemistry and Materials Science, and National Synchrotron Radiation Laboratory, University of Science and Technology of China, Hefei 230026, China; State Key Laboratory of Loess and Quaternary Geology (SKLLQG), Key Laboratory of Aerosol Chemistry and Physics, Institute of Earth Environment, Chinese Academy of Sciences, Xi’an 710061, China; Center of Excellence in Quaternary Science and Global Change, Chinese Academy of Sciences, Xi’an 710061, China; State Key Laboratory of Loess and Quaternary Geology (SKLLQG), Key Laboratory of Aerosol Chemistry and Physics, Institute of Earth Environment, Chinese Academy of Sciences, Xi’an 710061, China; Center of Excellence in Quaternary Science and Global Change, Chinese Academy of Sciences, Xi’an 710061, China; Institute of Atmospheric Physics, Chinese Academy of Sciences, Beijing 100190, China; School of Physics and Information Technology, Shaanxi Normal University, Xi’an 710062, China; Hefei National Research Center for Physical Sciences at the Microscale, School of Chemistry and Materials Science, and National Synchrotron Radiation Laboratory, University of Science and Technology of China, Hefei 230026, China

**Keywords:** sustainable CO_2_ utilization, artificial photosynthesis, all-weather application, charge storage

## Abstract

Solar-driven CO_2_ conversion into hydrocarbon fuels is a sustainable approach to synchronously alleviating the energy crisis and achieving net CO_2_ emissions. However, the dependence of the conversion process on solar illumination hinders its practical application due to the intermittent availability of sunlight at night and on cloudy or rainy days. Here, we report a model material of Pt-loaded hexagonal tungsten trioxide (Pt/h-WO_3_) for decoupling light and dark reaction processes, demonstrating the sustainable CO_2_ conversion under dark conditions for the first time. In such a material system, hydrogen atoms can be produced by photocatalytic water splitting under solar illumination, stored together with electrons in the h-WO_3_ through the transition of W^6+^ to W^5+^ and spontaneously released to trigger catalytic CO_2_ reduction under dark conditions. Furthermore, we demonstrate using natural light that CH_4_ production can persist at night and on rainy days, proving the accomplishment of all-weather CO_2_ conversion via a sustainable way.

## Teaser

The solar-driven CO_2_ reduction is free from the uncertainty of sunlight supply, allowing all-weather CO_2_ utilization.

## INTRODUCTION

Solar-driven CO_2_ conversion into CO, CH_4_, CH_3_OH and other products through photocatalysis offers a sustainable approach to synchronously alleviating the energy crisis and achieving net CO_2_ emissions [1–5]. However, since the lifetime of photogenerated electrons is usually in the order of sub-picoseconds to seconds [6,7], the photocatalytic reaction will stop rapidly upon the end of illumination. The asynchrony between solar energy supply and utilization demand, affected by day length and weather, is a great obstacle for the practical application of CO_2_ photoreduction [8,9]. As such, it is of great significance to develop a method for decoupling CO_2_ reduction from solar energy supply toward the goal of round-the-clock and all-weather CO_2_ conversion.

**Figure 1.**
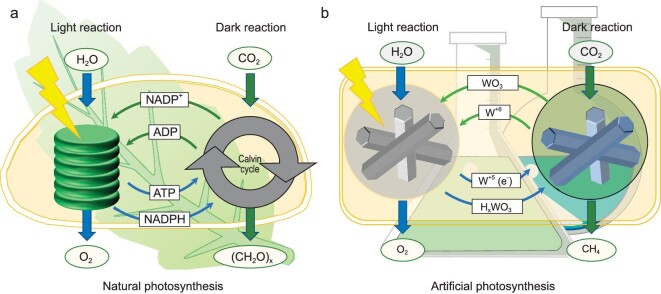
(a) The decoupled light and dark reaction process of natural photosynthesis. (b) The decoupled light and dark reaction process of artificial photosynthesis in this work.

Natural photosynthesis offers us an insight into the decoupling of light and dark reactions. The conversion of CO_2_ into carbohydrates by natural photosynthesis in green plants is divided into two steps—the widely known light reaction and dark reaction. During the light reaction, chloroplasts can synthesize reduced equivalents, which are used to produce the reduced nicotinamides adenine dinucleotide phosphate (NADPH) and adenosine triphosphate (ATP) (Fig. 1a) [10]. In the dark reaction, with the help of NADPH and ATP, CO_2_ can be converted stepwise to generate carbohydrates. Pioneering studies have proposed the multistep solar-derived hydrogen production. In a typical example, Amthor *et al.* used a covalent photosensitizer–polyoxometalate dyad to store the photogenerated electrons in polyoxometalate under visible-light irradiation [11]. Subsequently, hydrogen can be released by adding a proton donor to the dyad solution in an on-demand manner. In another case, Lau *et al.* reported the formation of ‘blue radical’ species within the cyanamide-functionalized polymeric network of heptazine units under solar irradiation [12]. By adding a Pt cocatalyst, the ‘blue radical’ species can give off the trapped electrons in the dark to release H_2_.

While the delayed on-demand solar hydrogen production has been achieved by several seminal works [11–13], it still remains a challenge to decouple light and dark reactions for CO_2_ conversion, particularly toward hydrocarbon fuel production, as the process involving multielectron hydrogen-coupled reduction is substantially more complex than H_2_ production. To make it work for such a complex process, both the photogenerated electrons and the hydrogen atoms have to be efficiently stored in a well-designed material under light irradiation so that the CO_2_ reduction process can then be triggered spontaneously upon their release in the dark. In principle, the reduction of CO_2_ to CH_4_ through a proton/electron pathway undergoes a negative change in Gibbs energy (ΔG° = –113 kJ mol^–1^) (Supplementary Fig. S1) [14]. It is thus feasible to achieve sustainable CO_2_ methanation in the dark, which is decoupled from the solar irradiation that provides the reduced protons and electrons.

To achieve the decoupling of the light reaction and dark reaction processes, the catalyst has to meet stringent requirements. First, under solar illumination, photocatalytic water splitting should occur to produce hydrogen atoms and electrons for energy storage. Second, the catalyst should possess suitable sites for storing hydrogen atoms and electrons. Third, driving forces should be present within the catalyst to facilitate the storage of electrons and hydrogen atoms under illumination as well as their subsequent release in the dark, enabling the reaction to take place spontaneously. Moreover, the structural changes during the storage and release of electrons and hydrogen atoms should be reversible to ensure cyclic stability. Among various catalytic materials, hexagonal tungsten oxide (h-WO_3_)-based photocatalysts can perfectly meet these specific requirements. Specifically, the energy band position of engineered h-WO_3_ can meet the needs of photocatalytic CO_2_ conversion and water splitting to product hydrogen atoms and electrons [15,16], despite its relatively weak reduction capacity. In addition, h-WO_3_ has a rich channel structure [17], providing abundant sites for hydrogen storage. Furthermore, in the presence of suitable cocatalysts (e.g. Pt, Pd, Cu), the hydrogen spillover process can be realized, which allows a fraction of hydrogen atoms to be transferred to the storage sites instead of being released in the form of H_2_ [18]. In principle, the processes of storing and releasing H atoms in h-WO_3_ are accompanied by reversible transformation of W^5+^ and W^6+^ to maintain charge balance and, as such, no structural damage of h-WO_3_ occurs [19]. Taken together, the modified h-WO_3_ is an ideal model material for demonstrating the concept of decoupling the light reaction and dark reaction processes for all-weather CO_2_ utilization.

Here, we report a well-designed material model for decoupling the light reaction and dark reaction to realize CO_2_ utilization in the dark. As illustrated in Fig. 1b, Pt-loaded h-WO_3_ (noted as Pt/h-WO_3_) is employed as a model catalyst to demonstrate our concept. In the light reaction, the light irradiation on the catalyst initiates photocatalytic water splitting, which in turn stores some of the electrons and H atoms in h-WO_3_. Upon the end of illumination, the stored electrons and H atoms are spontaneously released from the h-WO_3_ to trigger the catalytic reduction of CO_2_ in the dark. On the advantages of decoupling light and dark reactions, this work opens a new avenue for sustainable round-the-clock and all-weather CO_2_ utilization.

## RESULTS AND DISCUSSION

### Material synthesis and characterization

**Figure 2.**
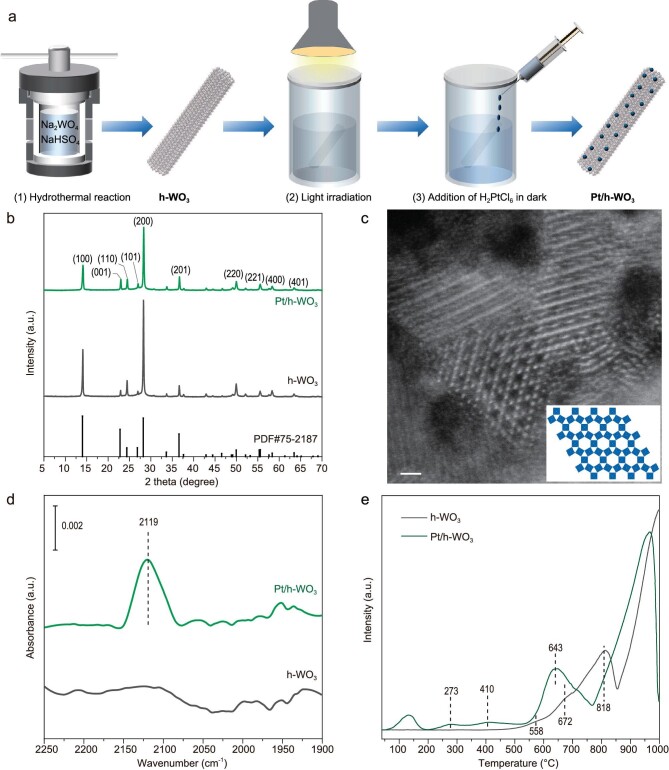
(a) Schematic diagram of catalyst synthesis. (b) XRD patterns of Pt/h-WO_3_ and h-WO_3_. (c) Representative HAADF-STEM image and projections of the structure along the *c*-axis (inset). Scale bars: 1 nm. (d) FTIR spectra after CO adsorption on h-WO_3_ and Pt/h-WO_3_, respectively. (e) H_2_-TPR spectra of h-WO_3_ and Pt/h-WO_3_.

Pt/h-WO_3_ was prepared by hydrothermal synthesis of h-WO_3_ followed by *in situ* deposition of Pt, as illustrated in Fig. 2a. The synthesized sample exhibits a nanorod-like structure with diameters of between 300 and 500 nm (Supplementary Figs S2 and S3). The diffraction peaks of X-ray diffraction (XRD) patterns show that the synthesized sample contains h-WO_3_ (PDF#75-2187) (Fig. 2b). The hexagonal structure is based on an arrangement of WO_6_ octahedra sharing corners in (WO_6_)_6_ wheels, which are stacked along the *c*-axis to yield hexagonal tunnels (Supplementary Fig. S4) [19]. The existence of hexagonal tunnels was proven by using atomic resolution aberration-corrected high-angle annular dark-field scanning transmission electron microscopy (HAADF-STEM) (Fig. 2c), which is consistent with the N_2_ sorption isotherms results (Supplementary Fig. S5). According to previous reports [19,20], the tunnel structure of Pt/h-WO_3_ is conducive to species embedding and dissociation, which is beneficial for the storage of hydrogen species.

In our synthesis, Pt modification was achieved through *in situ* reducing Pt^4+^ with the photogenerated electrons stored in h-WO_3_. The successful addition of Pt to h-WO_3_ nanorods can be demonstrated by using high-resolution Pt 4f X-ray photoelectron spectroscopy (XPS) (Supplementary Fig. S6) and the Pt loading content of Pt/h-WO_3_ is 0.11 wt% as measured by using inductively coupled plasma-mass spectrometry (ICP–MS). Note that no peaks corresponding to Pt species are found in XRD patterns, suggesting that Pt atoms are not in a crystalline form. To look into the form of Pt atoms, diffuse reflectance Fourier transform infrared (FTIR) spectroscopy was employed to examine the sample using CO as a probe molecule. As shown in Fig. 2d, Pt/h-WO_3_ displays a CO adsorption peak at 2119 cm^−1^, which is different from the adsorption of CO on Pt nanoparticles (2080 cm^−1^) (Supplementary Fig. S7), indicating that Pt was mainly loaded on the surface of Pt/h-WO_3_ in the form of highly dispersed atoms [21]. Moreover, the corresponding energy-dispersive spectroscopy mapping for Pt/h-WO_3_ confirmed that the Pt atoms are homogeneously distributed on the Pt/h-WO_3_ catalyst (Supplementary Fig. S8). Upon the addition of Pt, the absorption of Pt/h-WO_3_ in the visible range is slightly reduced (Supplementary Fig. S9), which is mainly caused by the decrease in W^5+^ content in the sample [15]. Further characterization shows that Pt can effectively accept electrons from the conduction band of the h-WO_3_ carrier so that the photogenerated charges are efficiently separated and in turn localized on the catalyst surface (Supplementary Figs S10–S13). The above characterizations prove that we have successfully introduced Pt to the surface of h-WO_3_, which can effectively improve the utilization rate of photogenerated charges.

Another consideration for integrating h-WO_3_ with Pt is to potentially utilize hydrogen spillover for harnessing hydrogen storage into h-WO_3_. The migration of H atoms from Pt to the tungsten trioxide was reported by Khoobiar as early as 1964 [22]. To this end, we studied the hydrogen spillover phenomenon over Pt/h-WO_3_ by using H_2_ temperature programmed reduction (H_2_-TPR). As shown in Fig. 2e, the three reduction peaks all move to a low temperature after Pt is deposited onto the h-WO_3_, suggesting the hydrogen spillover from Pt sites to the h-WO_3_ carrier [18]. Moreover, previous reports revealed that water could significantly increase the diffusion rate of the reducing species from Pt to tungsten trioxide [23]. In our case, once light irradiation produces H atoms from water with the photogenerated electrons, it would be feasible to insert H atoms into the h-WO_3_ carrier with a tunnel structure in aqueous solution.

### Catalytic CO_2_ reduction performance

**Figure 3.**
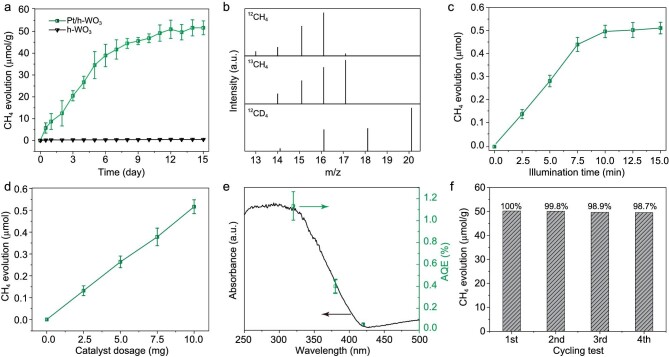
(a) CH_4_ evolution on Pt/h-WO_3_ and h-WO_3_ under dark conditions after 10 min of simulated sunlight irradiation. (b) Mass spectra of product CH_4_ from the photocatalytic reaction of ^12^CO_2_ (top) and ^13^CO_2_ (middle) with H_2_O over Pt/h-WO_3_, and CD_4_ from the photocatalytic reaction of ^12^CO_2_ with D_2_O (bottom). (c) Yield of CH_4_ from the reaction in the dark after different time-of-light irradiations. (d) Yield of CH_4_ over catalyst with different dosage under dark conditions after 10 min of simulated sunlight irradiation. (e) Wavelength-dependent AQE of CO_2_ reduction to CH_4_ on Pt/h-WO_3_. (f) Stability of Pt/h-WO_3_ during four photocatalytic cycles. Error bars denote the standard deviation of data from three tests.

Upon proposing that the synthesized material may store electrons and hydrogen atoms, we are now in a position to prove whether the stored energy can be used to reduce CO_2_ under dark conditions. To this end, we analysed the band structure of Pt/h-WO_3_ and found that it can meet the demand of CO_2_ reduction (Supplementary Fig. S9). The CO_2_ reduction performance of catalysts was then investigated using a home-made reactor (Supplementary Fig. S14). During the measurements, the catalysts were charged in pure water under simulated solar illumination for 10 min, followed by a CO_2_ reduction reaction under dark conditions. After light illumination, a certain amount of O_2_ and H_2_ were detected (Supplementary Fig. S15), indicating that photocatalytic water splitting had occurred. The ratio of detected H_2_ production to O_2_ production was dramatically less than the stoichiometric ratio of water splitting (2 : 1), indicating that a fraction of H atoms were stored [24]. Given the storage of H atoms during the 10 min of light illumination, high-purity CO_2_ was then injected into the reactor for a reduction reaction in the dark. As shown in Fig. 3a, after 10 min of light illumination, the catalytic CO_2_ reduction reaction continued for 10 days after the light was turned off and the yield of CH_4_ reached 51.6 μmol/g. The CH_4_ yield is equivalent to 309.6 μmol/g per hour of illumination, which was a fairly high rate in pure water systems compared with most of the existing reports (Supplementary Table S1). No other carbon-containing products were detected except for a small amount of CO (Supplementary Fig. S16). As Pt is an excellent cocatalyst for hydrogen generation, H_2_ was detected in the product after the reaction (Supplementary Fig. S17). Even without CO_2_, H_2_ can be released under dark conditions (Supplementary Fig. S18). To suppress the H_2_ evolution, we further increased the pressure of the reaction system. As a result, the mass transfer process of CO_2_ conversion could be improved by effectively turning down the process of H_2_ evolution [25], in which the yield of CH_4_ was further increased from 51.6 to 72.0 μmol/g (Supplementary Fig. S19). In addition, we found that the products can be controlled by modifying the cocatalyst, such as replacing Pt with Cu, and the main products changed to H_2_ (Supplementary Fig. S20). In comparison, when CO_2_ was replaced by high-purity Ar in the dark reaction process, only a negligible amount of CH_4_ was detected (0.21 μmol/g), indicating that CH_4_ is predominately generated from the CO_2_ in the dark reaction [26]. The results of the ^13^C isotope labeling experiment confirm that the CH_4_ in the product really originated from CO_2_ reduction rather than carbon impurities (Fig. 3b). In addition, the reaction product changed to CD_4_ by replacing H_2_O with D_2_O, proving that the hydrogen in the product came from water. From the above results, we can safely conclude that the decoupling of light and dark reactions indeed achieves CO_2_ reduction in the dark.

In order to verify that the reduction products originated from the stored energy in the catalyst, we carried out a series of control experiments. First of all, CO_2_ was directly introduced into the reactor in the absence of light irradiation and no carbon-containing products were generated, indicating that the driving force of CO_2_ reduction comes from light. Thus, we explored the influence of the light-irradiation time on the amount of CH_4_ generation. As shown in Fig. 3c, the product of CH_4_ increases by prolonging the illumination time before the dark reaction, suggesting that CO_2_ conversion depends on the number of photons. However, when the light time was extended to >10 min, the increase in CH_4_ yield was very limited, indicating that saturation can be reached after 10 min of illumination. To further prove whether the energy is stored in the catalyst, different dosages of catalysts were used to carry out CO_2_ reduction tests. As shown in Fig. 3d, the production of CH_4_ in the dark reaction was gradually promoted with an increase in the catalyst used, suggesting that the driving force for CO_2_ reduction is stored in the catalyst during the illumination process. When the catalyst dosage was increased by two orders of magnitude, the production rate of CH_4_ per unit mass catalyst was almost maintained (Supplementary Fig. S21), indicating that the catalyst has the prospect for further large-scale application. In addition, we expressed the efficiency of energy storage in terms of the apparent quantum efficiency (AQE). The efficiency of photon-to-chemical energy conversion under different monochromatic light wavelengths is summarized in Fig. 3e. The AQE of 1.13% at 320 nm demonstrates that short-wavelength light excites the catalyst. The information gleaned above demonstrates that the reduction of CO_2_ under dark conditions is triggered by the energy from light stored in the catalyst.

The catalyst stability is another critical factor that largely determines whether the catalyst can be used in practice. The cyclic stability test indicates that the recycled catalyst retains ∼98.7% of the original activity after four runs (Fig. 3f). The stability of the catalyst was also proven by the XRD patterns, FTIR spectra and XPS spectra of the catalyst after the reaction in comparison with the fresh one (Supplementary Fig. S22). The content of Pt after cycle testing was measured by using ICP–MS, revealing that the content of Pt (0.11 wt%) did not change during the reaction, which demonstrates the good stability of the material. In addition, the concentration of oxygen vacancies did not change significantly during the whole reaction, indicating that the structure of the catalyst was relatively stable during the reaction (Supplementary Fig. S23). In all, our designed Pt/h-WO_3_ can decouple the light and dark reaction processes to achieve CO_2_ reduction under dark conditions with high recyclability, indicating that such a working mechanism meets the preliminary requirement for sustainable all-weather CO_2_ conversion.

### Mechanism of decoupled CO_2_ conversion process

The successful practice, in which the Pt/h-WO_3_ material with light-irradiation pretreatment can sustain CO_2_ conversion under dark conditions, urges us to decode the mechanism of decoupling light and dark reaction processes with systematic investigations. As such, we have extensively examined the mechanisms for energy storage under light irradiation and energy release in the dark. During the whole reaction process, the most distinct phenomenon was the significant color change of the catalyst. The color of the catalyst changed from gray white to light blue in the process of illumination but slowly returned to its original color after the dark reaction (Supplementary Fig. S24). This phenomenon is related to the change of light absorption in the visible-light range. Visible or near infrared light can induce polaron transitions—the hopping of polarons from W^5+^ to nearby W^6+^ positions [15], resulting in light absorption. In our case, W^5+^ was generated in the process of illumination and consumed in the dark reaction, altering the light absorption.

**Figure 4.**
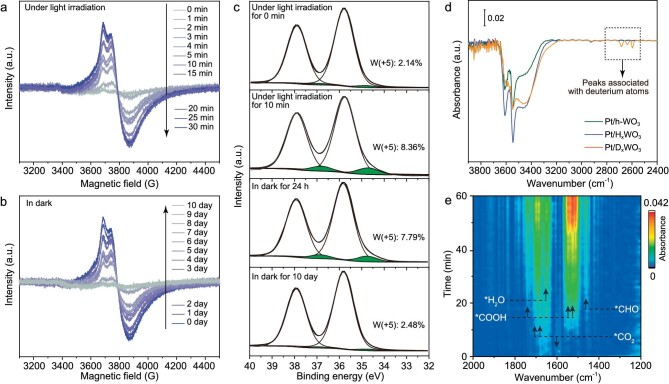
EPR spectra of Pt/h-WO_3_ (a) under light irradiation and then (b) in the dark. (c) High-resolution W 4f XPS spectra of Pt/h-WO_3_ before reaction, after light reaction, after dark reaction for 24 h and for 10 days, respectively. (d) FTIR spectra of pristine Pt/h-WO_3_ and Pt/h-WO_3_ after light reaction using H_2_O (noted as Pt/H_x_WO_3_) and D_2_O (noted as Pt/D_x_WO_3_), respectively. (e) *In situ* FTIR spectra in the dark over Pt/h-WO_3_ after light reaction.

To look into the generation and consumption of W^5+^, electron paramagnetic resonance (EPR) spectroscopy was employed to examine the catalyst after light and dark reactions. The EPR signal of W^5+^ was characterized by using an axial g-tensor with g values of 1.909 and 1.880 [19,27]. Under the simulated solar light, the EPR signal for W^5+^ appeared and reached the maximal intensity after 10 min (Fig. 4a). After turning off the illumination, the intensity of the EPR signal related to W^5+^ in the catalyst slowly decreased and finally returned to the original state (i.e. prior to the illumination) after 10 days (Fig. 4b). The consistency between the evolution of W^5+^ species and the process of CO_2_ reduction in the timescale indicates that the CO_2_ reduction under dark conditions should have involved W^5+^ species. To further elucidate the origin and destination of W^5+^, we studied the valence state of W at different reaction stages by using XPS. As shown in Fig. 4c, the deconvoluted W 4f spectrum of Pt/h-WO_3_ can be fitted into two W oxidation states, namely W^6+^ (4f_7/2_, 35.82 eV) and W^5+^ (4f_7/2_, 34.81 eV), without other valences [15,28,29]. The ratio of W^5+^ in the Pt/h-WO_3_ was promoted from 2.14% to 8.36% after the light reaction, suggesting that a part of the W^6+^ was reduced to W^5+^ in the process of illumination. Such a reduction of W^6+^ to W^5+^ during illumination is essentially a process of storing the photogenerated electrons. In the dark reaction process, the proportion of W^5+^ slowly decreased to 7.79% after 24 h and returned to the initial state (i.e. 2.48%) after 10 days. This manifests the dark reaction process that the stored electrons were spontaneously released after turning off the illumination, which in turn triggered the CO_2_ reduction reaction.

With the information for electron storage in mind, we are still questioned by two remaining issues—the source of H in the CH_4_ product and the destination of the positive charge species along with the electron storage in W^5+^. As we originally proposed, both should be associated with the formation of H atoms from photocatalytic water splitting and their insertion into the catalyst. To further verify that H atoms had inserted into h-WO_3_ during the light reaction, we characterized the Pt/h-WO_3_ before and after the light reaction by using FTIR spectroscopy. Note that, due to the presence of a small amount of water and hydrogen atoms in the catalyst (Supplementary Fig. S25 and Supplementary Table S2), we can only judge whether additional hydrogen atoms are inserted into the catalyst by the change in the intensity of the peak. After the light reaction, the peaks attributed to the O–H species of ∼3500–3700 cm^−1^ were obviously enhanced, indicating that the content of H in the sample had increased (Fig. 4d). Further experiments were carried out by replacing the water (H_2_O) with deuterium oxide (D_2_O). The spectra showed that the FTIR peak attributed to the O–D species appeared at 2500–2700 cm^−1^ after the light irradiation while the peaks for the O–H species of ∼3500–3700 cm^−1^ were fairly similar to those of pristine Pt/h-WO_3_, indicating that the H that was inserted into the catalyst came from water splitting. In addition, we further quantified the stored hydrogen by means of ion exchange (Supplementary Table S2). The above characterization indicates that H was inserted and stored in the Pt/h-WO_3_ during the light reaction process.

Next, we further proved whether the stored H atoms and electrons can trigger CO_2_ reduction under dark conditions. To this end, *in situ* FTIR spectroscopy was performed to reveal the activation of the CO_2_ by the stored H atoms and electrons. For fresh Pt/h-WO_3_, after being exposed to CO_2_/H_2_O vapors in the dark, the peaks at 1700, 1688 and 1603 cm^−1^ that should be attributed to the formation of bidentate carbonate b-CO_3_^2−^ appeared [30,31] (Supplementary Fig. S26)_._ In addition, the peak for adsorbed H_2_O was also detected at 1641 cm^−1^ [15]. No intermediates corresponding to CO_2_ methanation could be detected. In contrast, after Pt/h-WO_3_ has been treated with H_2_O vapor under light irradiation, some peaks attributed to intermediates can be detected upon exposure to CO_2_/H_2_O vapors in the dark (Fig. 4e). Most notably, the absorption peaks attributed to COOH intermediates were observed at 1730, 1580 and 1547 cm^−1^, respectively [30,32–34]. Previous studies have reported that the formation of the *COOH structure is a crucial step in CO_2_ activation [30,33]. Thus, this observation indicates that the H atoms and electrons stored in Pt/h-WO_3_ can trigger CO_2_ activation under dark conditions. In addition, the absorption peak attributed to *CHO was also observed (at 1460 cm^−1^), suggesting that CO_2_ followed the path of CH_4_ formation [35,36].

Based on the above characterization results, the light and dark reactions are expressed by the following equations:

Light reaction: (1)


\begin{eqnarray*}
&& {\mathrm{Pt}}/{\mathrm{W}}{{\mathrm{O}}}_3 ( {6 + }) + \frac{{\mathrm{x}}}{2}{{\mathrm{H}}}_2{\mathrm{O}}\mathop \to \limits^{{\mathrm{hv}}} {\mathrm{Pt}}/{{\mathrm{H}}}_{\mathrm{x}}{\mathrm{W}}{{\mathrm{O}}}_3\left( {5 + } \right) \\
&&\quad +\, \frac{\rm x}{4}{{\mathrm{O}}}_2
\end{eqnarray*}

Dark reaction: (2)


\begin{eqnarray*}
&& {\mathrm{Pt}}/{{\mathrm{H}}}_{\mathrm{x}}{\mathrm{W}}{{\mathrm{O}}}_3 ( {5 + } ) + \frac{{\mathrm{x}}}{8}{\mathrm{C}}{{\mathrm{O}}}_2 \to {\mathrm{Pt}}/{\mathrm{W}}{{\mathrm{O}}}_3 ({6 + }) \\
&&\quad +\, \frac{{\mathrm{x}}}{8}{\mathrm{C}}{{\mathrm{H}}}_4+ \frac{\rm x}{4}{{\mathrm{H}}}_2{\mathrm{O}}
\end{eqnarray*}

In the light reaction process, the electrons are excited from the valence band of the h-WO_3_ carrier to its conduction band and then transferred to the Pt sites to realize the water splitting. The O atoms are oxidized by the holes remaining in the valence band to release O_2_, while the H atoms at the Pt site spill over the h-WO_3_ carrier. In the meantime, a part of W^6+^ in the h-WO_3_ surface is reduced to W^5+^ for electron storage. In the dark reaction, the stored electrons and H atoms are spontaneously released to achieve CO_2_ reduction.

Overall, the unique performance of our designed material, with the capability of storing and releasing energy in light on/off cycles, should be related to the fact that the H atom, as an energy carrier, can be stored in the channel of h-WO_3_ via the spillover through Pt. To further illustrate this point, we highlight the importance of two processes—the spillover and storage of H atoms along with electrons, and the release of H atoms and electrons, with reference samples separately. First, we prepared Au-loaded h-WO_3_ as a reference sample and found that no hydrogen spillover takes place on this catalyst (Supplementary Fig. S27). As a result, the CO_2_ conversion performance is nearly equal to that of bare h-WO_3_. This indicates that the effective hydrogen spillover to the h-WO_3_ carrier is a prerequisite for CO_2_ conversion in the dark. Second, the reference sample of Ni-loaded h-WO_3_ offers the ability of storing H atoms and electrons along with H spillover (Supplementary Fig. S28). However, under the dark conditions, the stored electrons and H atoms cannot be released spontaneously, hindering the CO_2_ reduction. In comparison, the reference sample of Pd-loaded h-WO_3_ possesses similar properties to Pt/h-WO_3_, in which the electrons and H atoms can be both stored and released, enabling CO_2_ conversion under dark conditions (Supplementary Fig. S29). Taken together, the above reference samples clearly demonstrate that the efficient storage and spontaneous release of electrons and H atoms together ensure the decoupling of light and dark reactions for CO_2_ conversion.

### Demonstration of sustainable solar-driven application

**Figure 5.**
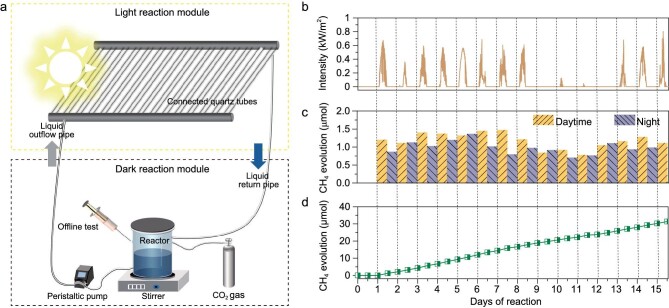
(a) Schematic diagram of outdoor experimental equipment. (b) Solar light intensity. (c) Production of CH_4_ in 16 consecutive days with 12-h daytime and 12-h night-time. Daytime represents 7 : 00 to 19 : 00, and night-time represents 19 : 00 to 7 : 00 of the next day. (d) Cumulative yield of CH_4_.

In order to verify the feasibility of our concept in practical application, we conducted a round-the-clock and all-weather demonstration of CO_2_ conversion under natural light irradiation. We divided the reactor into a light reaction module and a dark reaction module (Fig. 5a and Supplementary Figs S30 and S31). The light reaction module was designated for sunlight absorption and energy storage, and then the CO_2_ reduction reaction was conducted in the dark reaction module. The light reaction module and dark reaction module were connected through pipes and the catalyst dispersion in pure water was driven by using a peristaltic pump to realize circulation in the two modules. The experiment was conducted from 8 to 23 September 2022 in Xi’an, China. We recorded the light intensity during this period by using a solar radiometer. The specific weather conditions are listed in Supplementary Table S3. As shown in Fig. 5b, the solar light intensity decreases significantly on cloudy days and decreases to zero at night and on rainy days.

On the first day of the reaction, we put the whole reaction system in the dark. We found that no CH_4_ was generated (Fig. 5c and d), indicating that the energy for the subsequent generation of CH_4_ was all from sunlight. On the second day of the experiment, the reaction system was transferred outdoors. CH_4_ was slowly generated under sunlight, with a CH_4_ yield of 1.2 μmol in the daytime. As it switched to the night, the CH_4_ production continued but was observed with a slightly reduced rate, which yielded another 0.86 μmol of CH_4_. This result demonstrates that we have successfully achieved round-the-clock CO_2_ reduction. The third day was cloudy so that the supply of sunlight decreased significantly; however, the rate of CH_4_ production increased slightly, indicating that the intermittent supply of energy was also collected. The fourth to ninth days were sunny with a certain amount of cloud cover and, as a result, the rate of CH_4_ production increased slowly and then reached an equilibrium. It is worth mentioning that, on the ninth to thirteenth days of the test, the rainy weather made the intensity of the Sun drop to near zero, but the CH_4_ production did not stop. Moreover, the performance of the catalyst recovered after exposure to solar irradiation. This result manifests that the unique reaction was not significantly affected by short-term weather change, demonstrating the all-weather application.

## CONCLUSION

In summary, we prepared a Pt-loaded h-WO_3_ as a model catalyst that can store photogenerated electrons and hydrogen atoms under light irradiation for dark reaction CO_2_ conversion. The CH_4_ yield of this catalyst reached 51.6 μmol/g under dark conditions after 10 min of simulated solar illumination and could be maintained at 98.7% after four cycles of use. While there is still plenty of room to improve the conversion rate in the future, this work clearly demonstrates for the first time that the concept of decoupling light and dark reaction processes can work for sustainable solar-driven CO_2_ conversion. Our systematic investigations have proven that the reduction in CO_2_ under dark conditions is indeed triggered by the electrons and hydrogen atoms that are generated by photon energy during light irradiation and stored in the catalyst. The unique characteristics of the h-WO_3_ carrier that offer variable valence states and tunnel structures, along with the capability of Pt in splitting water and spilling hydrogen atoms over onto the h-WO_3_ surface, are key to achieving the decoupling of light and dark reactions for CO_2_ conversion. Toward practical applications, we conducted a demonstration using natural light that CH_4_ production persisted at night and on rainy days, indicating that our proposed concept can achieve round-the-clock and all-weather CO_2_ conversion. On the strength of decoupling the light reaction and dark reaction, this work contributes to the solar-driven conversion of CO_2_ into valuable products in a sustainable way.

## METHODS

### Materials preparation

To prepare the h-WO_3_ nanorods, 0.99 g of Na_2_WO_4_·2H_2_O and 1.19 g of NaHSO_4_·H_2_O were dissolved in 40 mL of deionized water with constant stirring. After stirring for 1 h, the mixture solution was then transferred into a 100-mL autoclave and heated in oven at 180°C for 24 h. After reaction, the precipitates were collected by using centrifugation and washed using deionized water and ethanol several times. The powder was obtained and dried in a vacuum at 70°C overnight (noted as h-WO_3_). Pt-modified h-WO_3_ was obtained by an *in situ* reduction of Pt by low-valence W^5+^. Briefly, 0.2 g of h-WO_3_ and 100 mL of water were added into a 200-mL quartz closed reactor and then ultrasonically treated for 30 min to form a uniform light-white mixture solution. After the solution mixture was purged by using Ar for 1 h, a 500-W xenon lamp was used as the light source to irradiate the solution for 1 h. The color of the mixture solution changed into light blue after 1 h of illumination, indicating that the reduced W^5+^ was formed. Then 10 mL of H_2_PtCl_6_·xH_2_O (2 mg/mL) was dropped into the obtained solution with constant stirring. After stirring for 1 min, the sample was separated by using centrifugation and washed three times alternately with deionized water and ethanol, and dried in a vacuum at 70°C overnight (noted as Pt/h-WO_3_).

### Catalytic CO_2_ reduction measurement

The CO_2_ reduction performance of the as-obtained samples was measured in a home-made cylindrical quartz closed reactor (200 mL). First, 10 mg of catalyst and 60 mL of deionized water were added into the reactor and then ultrasonically dispersed for 30 min. Second, the air in the reactor was removed by using a vacuum pump for 1 h. Then the simulated sunlight was produced by using a 300-W Xe arc lamp irradiated on the sample through a quartz window for a certain period of time. After illumination, a certain amount of high-purity CO_2_ (99.999%) was implanted into the reactor to keep the pressure equal to the atmosphere. Finally, the reactor was placed in the dark for the CO_2_ reduction reaction. The products were detected by using gas chromatography (7890B, Agilent) equipped with a flame ionization detector and a thermal conductivity detector. The reactor was maintained at 20°C by circulating water throughout the reaction. The AQE was calculated by using AQE (%) = (Number of evolved CH_4_ molecules × 8)/(Number of incident photons) × 100%. Using ^13^CO_2_ instead of ^12^CO_2_ or D_2_O instead of H_2_O as a reactant in the isotope labeling experiment, the product (CH_4_ or CD_4_) was detected by using gas chromatography-mass spectrometry (7890A and 5975C, Agilent).

## Supplementary Material

nwad275_Supplemental_File
