# Supplementary material for: Sustainable all-weather CO2 utilization by mimicking natural photosynthesis in a single material
Source: Natl Sci Rev. 2023 Oct 28;11(2):nwad275. doi: 10.1093/nsr/nwad275 (PMC10789249; doi:10.1093/nsr/nwad275)
Supplement: nwad275_Supplemental_File [file nwad275_supplemental_file.pdf]

Supplementary Information

Sustainable all-weather CO_2 utilization by mimicking natural photosynthesis in a single material

Xianjin Shi[1,2,3], Yu Huang[1,2,*], Ran Long[4], Zhenyu Wang[1,2], Liqin Wang[1,2], Junji Cao[5,*], Gangqiang Zhu[6] and Yujie Xiong[4,*]

[1]State Key Laboratory of Loess and Quaternary Geology (SKLLQG), Key Laboratory of Aerosol Chemistry and Physics, Institute of Earth Environment, Chinese Academy of Sciences, Xi'an 710061, China;

[2]Center of Excellence in Quaternary Science and Global Change, Chinese Academy of Sciences, Xi'an 710061, China;

[3]University of Chinese Academy of Sciences, Beijing 100049, China;

[4]Hefei National Research Center for Physical Sciences at the Microscale, School of Chemistry and Materials Science, and National Synchrotron Radiation Laboratory, University of Science and Technology of China, Hefei 230026, China;

[5]Institute of Atmospheric Physics, Chinese Academy of Sciences, Beijing 100190, China;

[6]School of Physics and Information Technology, Shaanxi Normal University, Xi'an 710062, China

*__Corresponding authors.__ E-mails: huangyu@ieecas.cn; jjcao@mail.iap.ac.cn; yjxiong@ustc.edu.cn

Experimental section

Materials

Chemicals. Sodium tungstate dihydrate ($Na_2WO_4 \cdot 2H_2O$, 99.5%), sodium bisulfate monohydrate ($NaHSO_4 \cdot H_2O$, 99.9%), chloroplatinic acid hydrate ($H_2Cl_6Pt \cdot xH_2O$) and deuterium water (D_2O) were purchased from Shanghai Macklin Biochemical Co., Ltd. Chloroauric acid trihydrate ($H_2AuCl_4 \cdot 3H_2O$), sodium tetrachloropalladate (II) (Na_2PdCl_4), nickel nitrate trihydrate ($Ni(NO_3)_2 \cdot 3H_2O$), copper nitrate trihydrate ($Cu(NO_3)_2 \cdot 3H_2O$) and sodium sulfate (Na_2SO_4) were purchased from Sinopharm Chemical Reagent Co., Ltd. All chemicals were used as received and without further purification. Deionized water used in all experiments is ultrapure water with a resistivity of 18.2 MΩ cm^{-1}.

Section 1. Preparation of h-WO_3

To prepare h-WO_3 nanorods, 0.99 g $Na_2WO_4 \cdot 2H_2O$ and 1.19 g $NaHSO_4 \cdot H_2O$ were dissolved in 40 mL deionized water with constant stirring. After stirring for 1 h, the mixture solution was then transferred into a 100 mL autoclave and heated in oven at 180 ℃ for 24 h. After reaction, the precipitates were collected by centrifugation, and washed with deionized water and ethanol for several times. The powder was obtained and dried in vacuum at 70 ℃ overnight (noted as h-WO_3).

Section 2. Preparation of Pt/h-WO_3

Pt-modified h-WO_3 was obtained by in situ reduction of Pt by low-valence W^{5+}. Briefly, 0.2 g h-WO_3 and 100 mL water were added into a 200 mL quartz closed reactor, and then ultrasonically treated for 30 min to form a uniform light-white mixture solution. After the solution mixture was purged by Ar for 1 h, a 500 W xenon lamp was used as the light source to irradiate the solution for 1 h. The color of the mixture solution changed into light blue after 1 hour of illumination, indicating that the reduced W^{5+} was formed. Then 10 mL of $H_2PtCl_6 \cdot xH_2O$ (2 mg/mL) was dropped into the obtained solution with constant stirring. After stirring for 1 min, the sample was collected and dried in vacuum at 70 ℃ overnight (noted as Pt/h-WO_3).

Section 3. Synthesis of h-WO_3 loaded with Pt nanoparticles

The h-WO_3 loaded with Pt nanoparticles (Pt NPs) was prepared by photo-deposition. Briefly, other conditions were the same as the preparation method of Pt/h-WO_3, except that the order of addition of $H_2PtCl_6 \cdot xH_2O$ and light irradiation was switched.

Section 4. Synthesis of Au-loaded h-WO_3

The preparation process of Au-loaded h-WO_3 was similar to that of Pt-loaded h-WO_3. Briefly, 0. 2 g h-WO_3 and 100 mL water were added into a 200 mL quartz closed reactor, and then ultrasonically treated for 30 min to form a uniform light-white mixture solution. After the solution mixture was purged by Ar for 1 h, a 500 W xenon lamp was used as the light source to irradiate the solution for 1 h. The color of the mixture solution changed into light blue after 1 hour of illumination, indicating that

the reduced W^{5+} was formed. Then 10 mL of $H_2AuCl_4 \cdot 3H_2O$ (2 mg/mL) was dropped into the obtained solution with constant stirring. After stirring for 1 min, the sample was collected and dried in vacuum at 70 ℃ overnight (noted as Au/h-WO_3). The Au loading content of Au/h-WO_3 is 0.15 wt% as measured by inductively coupled plasma-mass spectrometry (ICP-MS).

Section 5. Synthesis of Cu-loaded h-WO_3

The preparation method of Cu-loaded h-WO_3 (Cu/h-WO_3) was the same as that of Pt/h-WO_3, except that $H_2PtCl_6 \cdot xH_2O$ was replaced by $Cu(NO_3)_2 \cdot 3H_2O$. The Cu loading content of Cu/h-WO_3 is 0. 17 wt% as measured by ICP-MS.

Section 6. Synthesis of Ni-loaded h-WO_3

Since Ni is difficult to be reduced by W^{5+}, we extended the reaction time under dark conditions. Firstly, 0. 2 g h-WO_3 and 100 mL water were added into a 200 mL quartz closed reactor, and then ultrasonically treated for 30 min to form a uniform light-white mixture solution. After the solution mixture was purged by Ar for 1 h, a 500 W xenon lamp was used as the light source to irradiate the solution for 1 h. The color of the mixture solution changed into light blue after 1 hour of illumination, indicating that the reduced W^{5+} was formed. Then 10 mL of ($Ni(NO_3)_2 \cdot 3H_2O$) (2 mg/mL) was dropped into the obtained solution with constant stirring. After stirring for 24 h, the sample was collected and dried in vacuum at 70 ℃ overnight (noted as Ni/h-WO_3). The Ni loading content of Ni/h-WO_3 is 0.21wt% as measured by ICP-MS.

Section 7. General characterizations

XRD patterns were recorded on Panalytical X'pert Pro X-ray diffractometer with Cu Kα radiation (λ = 1.5406 Å, 40 kV, 40 mA). HAADF-STEM images were taken by Titan FEI-ETEM-G2 80-300 at an accelerating voltage of 300 kV. XPS spectra were collected on an X-ray photoelectron spectrometer (Thermo ESCALAB 250, USA). TPR was measured on a chemisorption analyzer (PCA 1200, BJbuilder, China). Steady-state photoluminescence (PL) spectroscopy (FLS980, Edinburgh, England) was employed to investigate the lifetime of the photo-induced charges and the optical properties of the resulting samples. Nitrogen adsorption-desorption isotherms and specific surface area were measured by gas adsorption analysis system (Quantachrome Autosorb-IQ, USA).

Section 8. Characterizations of EPR spectroscopy

EPR spectra were recorded on a ELEXSYS E500 spectrometer (Bruker, German) at room temperature. The reaction process was similar to that of CO_2 reduction measurement. After a certain time, a strong vacuum pump was used to exclude the water vapor above the solution, so that the water in the reaction chamber was quickly evaporated. After all the water was removed, the sample was transferred to a quartz tube (inner diameter 3 mm) under N_2 protection and the mouth of the tube was closed with a plug to avoid contact with air. Finally, the quartz tube was put into the instrument for EPR characterization.

Section 9. Characterizations of FTIR spectroscopy

FTIR characterization was performed on a VERTEX 70 FTIR spectrometer (Bruker, Germany) equipped with a Harrick in situ diffuse reflectance cell and liquid nitrogen cooled MCT (Mercury Cadmium Telluride) detector. For the CO adsorption, all the samples were preheated at 200 °C for 120 min to obtain a clean surface. The baseline for the spectra was recorded after cooling to room temperature. Subsequently, 1% CO/Ar was poured into the cell at 10 mL/min for 30 min. The cell was then purged with N_2 for 30 min to completely remove gaseous CO, and the FTIR spectrum was recorded at the end of the purging. For the in-situ CO_2 conversion measurement, after the light reaction, all the water was extracted by a mechanical pump. Subsequently, the catalyst was transferred to the in situ diffuse reflectance cell under nitrogen protection. Finally, 5% CO_2/N_2 with the flow rate of 10 mL/min was introduced through a container filled with water, and FTIR data was collected every 1 min in dark.

Section 10. Characterizations of light-assisted Kelvin probe force microscopy (KPFM)

Atomic force microscope (AFM) topography images and light-assisted Kelvin probe force microscopy (KPFM) were collected by AFM (Bruker MultiMode-8 surface potential mode) at ambient conditions. A SCM-PIT-V2 model tip and AS-130VLR ("J" vertical) scanner model was used, and the radius and elastic coefficient of tip

were 35 nm and 3 N/m, respectively. The tip lift height was 60 nm for potential mapping at tapping mode. A 300 W Xe arc lamp irradiated at the sample to provide light irradiation. In the process of testing, the samples were coated on highly oriented pyrolytic graphite (HOPG) substrate. The contact potential difference (CPD) was defined as the difference between the work function of the tip and the sample [1]. The images were processed by first order flattening to eliminate errors caused by sample tilt. The surface photovoltage (SPV) was calculated as SPV = ΔCPD = CPD_{dark} − CPD_{light}, where CPD_{dark} and CPD_{light} are the CPD measured in dark and under light illumination, respectively.

Section 11. Measurements of the amount of stored H

The amount of stored H was measured by means of ion exchange. 10 mg of photocatalyst powder was dispersed in 60 mL of deionized water and then ultrasonically dispersed for 30 min. Subsequently, the air in the reactor was removed by vacuum pump for 10 min while the pH of the solution was close to 7. Then the simulated sunlight was produced by a 300 W Xe arc lamp irradiated on the sample through quartz window for 10 min. After illumination, excessive KNO_3 solution (0.5 M, 4 mL) was dropped into the obtained solution in the dark with constant stirring. After stirring for 3 h until the pH no longer changed, the sample was separated by centrifugation, and the pH of the separated solution was measured. This value was used to evaluate the amount of released H through the following equation:

$$M(H) = 10^{-pH} \times 0.064 \times 10^{6}$$

where *M(H)* is the amount of hydrogen atoms (μmol).

Section 12. Characterizations of photoelectrochemical properties

The photoelectrochemical properties of the as-prepared samples were collected using a Parstat 4000 electrochemical workstation (USA) in a three-electrode cell. Pt foil and saturated Ag/AgCl electrode were used as the counter electrode and reference electrode, respectively. The potential plots were measured at the open-circuit voltage in 0.2 M Na_2SO_4 with phosphate buffer (pH = 7) as the electrolyte. The Mott–Schottky (M–S) plots were measured in the dark at fixed frequencies of 250, 500 and 750 Hz, respectively. Electrochemical impedance spectroscopy (EIS) was performed in 1 mM $K_3Fe(CN)_6$ and $K_4Fe(CN)_6$ solution. The photocurrent response spectra were measured at 0.2 V vs. Ag/AgCl in 0.5 M Na_2SO_3 at room temperature. Cyclic voltammetry (CV) of Pt/h-WO_3 in 0.1 M H_2SO_4 with a polished glassy carbon electrode (5 mm of diameter) as the working electrode. All solutions were purged with Ar or CO_2 before CV test.

Supplementary Figures

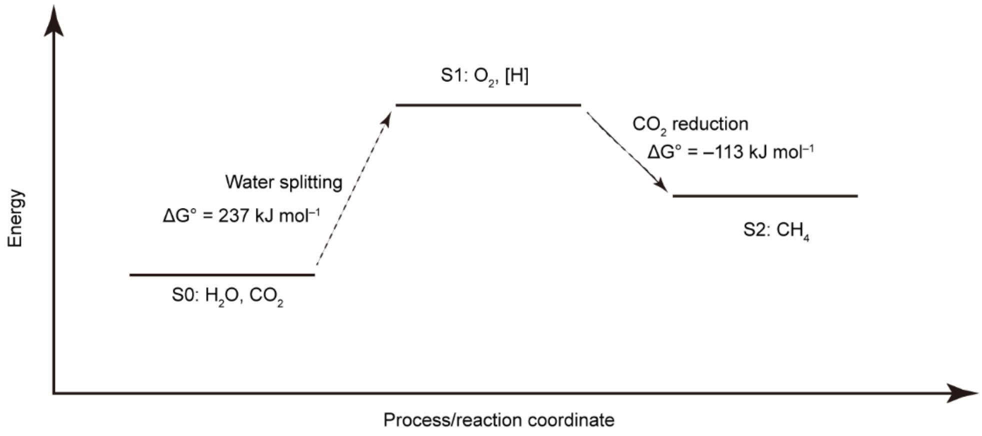

Supplementary Figure 1. Graphical representation of the thermodynamic states of the CO_2 methanation reaction [2].

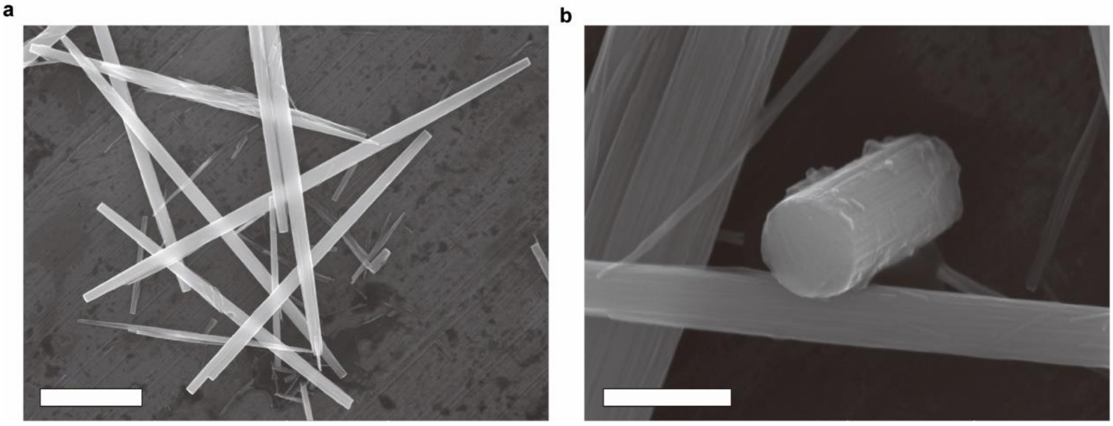

Supplementary Figure 2. Scanning electron microscopy (SEM) images of Pt/h-WO_3. Scale bars: 2 μm (a), 500 nm (b).

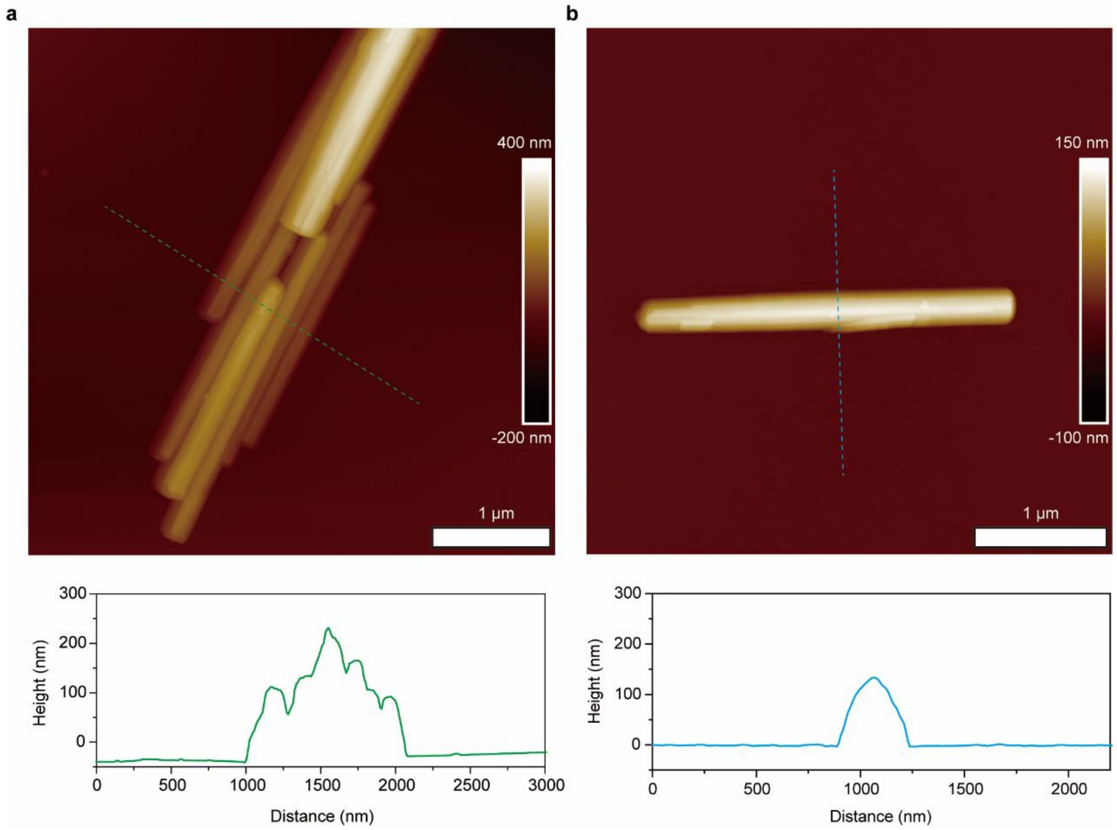

Supplementary Figure 3. AFM topography images (top) of Pt/h-WO_3 and the section profiles (bottom) along the line in topography image.

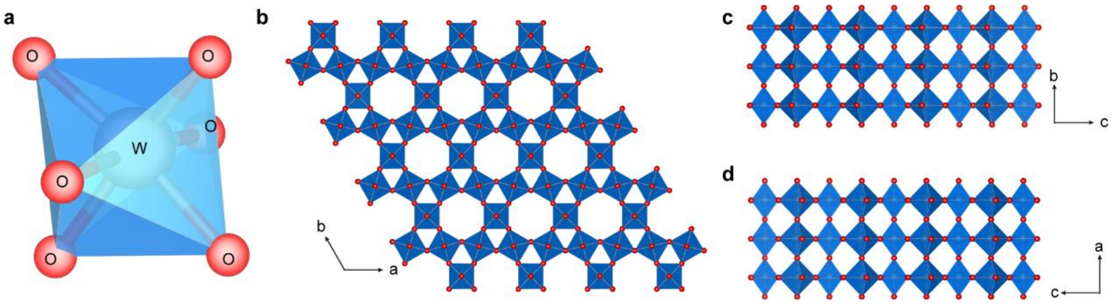

Supplementary Figure 4. The structure of hexagonal-phase WO_3. (a) WO_6 octahedra unit. Projections of the structure of h-WO_3 along the (b) c axis, (c) a axis, and(d) b axis.

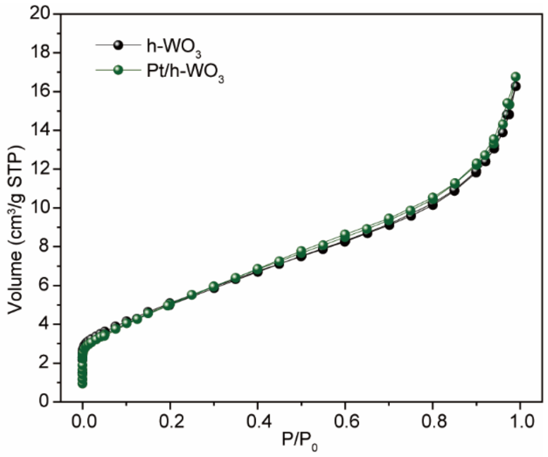

Supplementary Figure 5. N_2 sorption isotherms of h-WO_3 and Pt/h-WO_3.

The sharp increase at low relative pressure in N_2 adsorbed volume suggests the presence of microporosity, and is ascribed to the crystalline structure of h-WO_3 and Pt/h-WO_3 [3]. The specific surface area of h-WO_3 and Pt/h-WO_3 is 19.0 and 18.8 m^2/g, respectively, as calculated by the BET method.

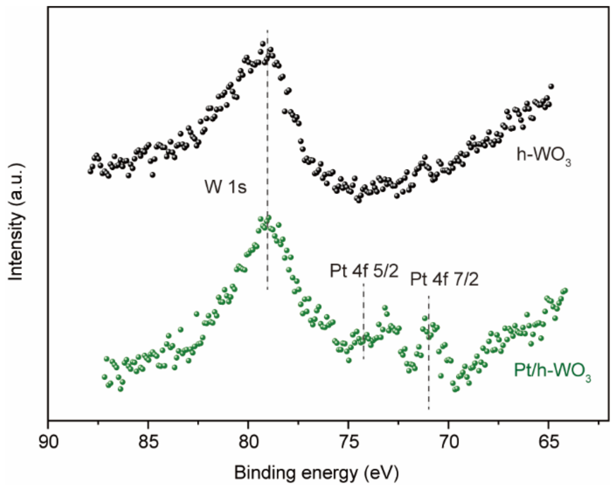

Supplementary Figure 6. High-resolution Pt 4f XPS spectra of h-WO_3 and Pt/h-WO_3.

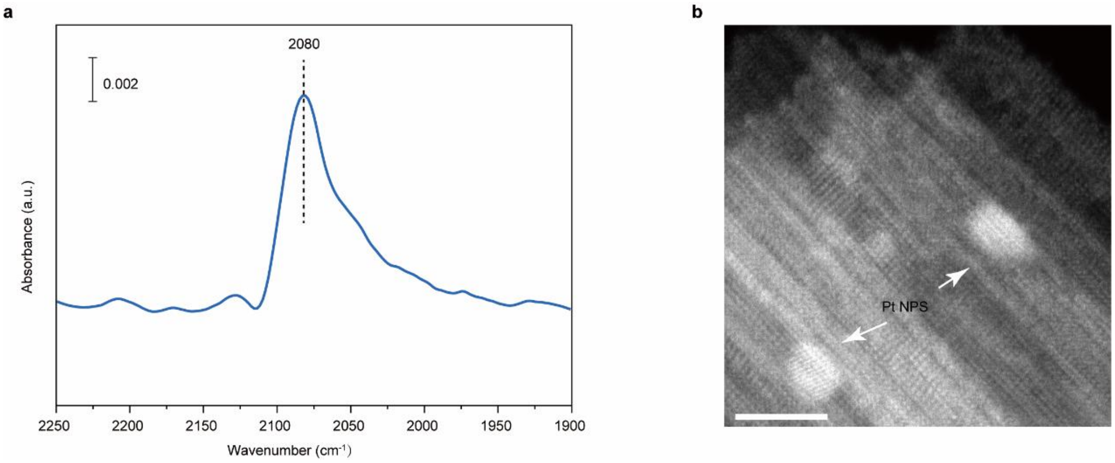

Supplementary Figure 7. (a) FTIR spectrum after CO adsorption on the h-WO_3 loaded with Pt NPs. (b) HAADF-STEM image of Pt NPs. Scale bar: 5 nm.

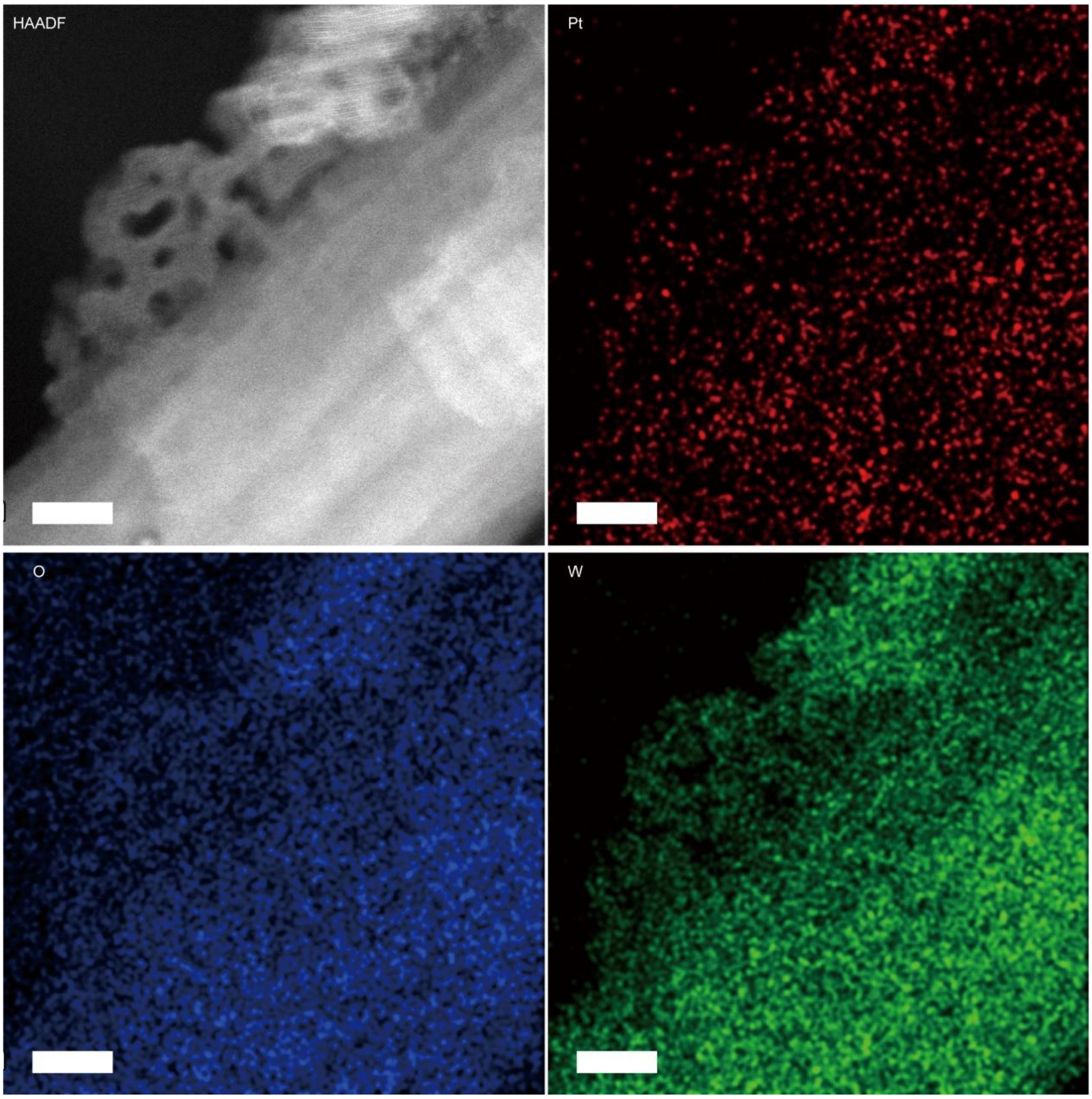

Supplementary Figure 8. Energy-dispersive spectroscopy (EDS) elemental mapping of Pt, W and O for Pt/h-WO_3. Scale bars: 5 nm.

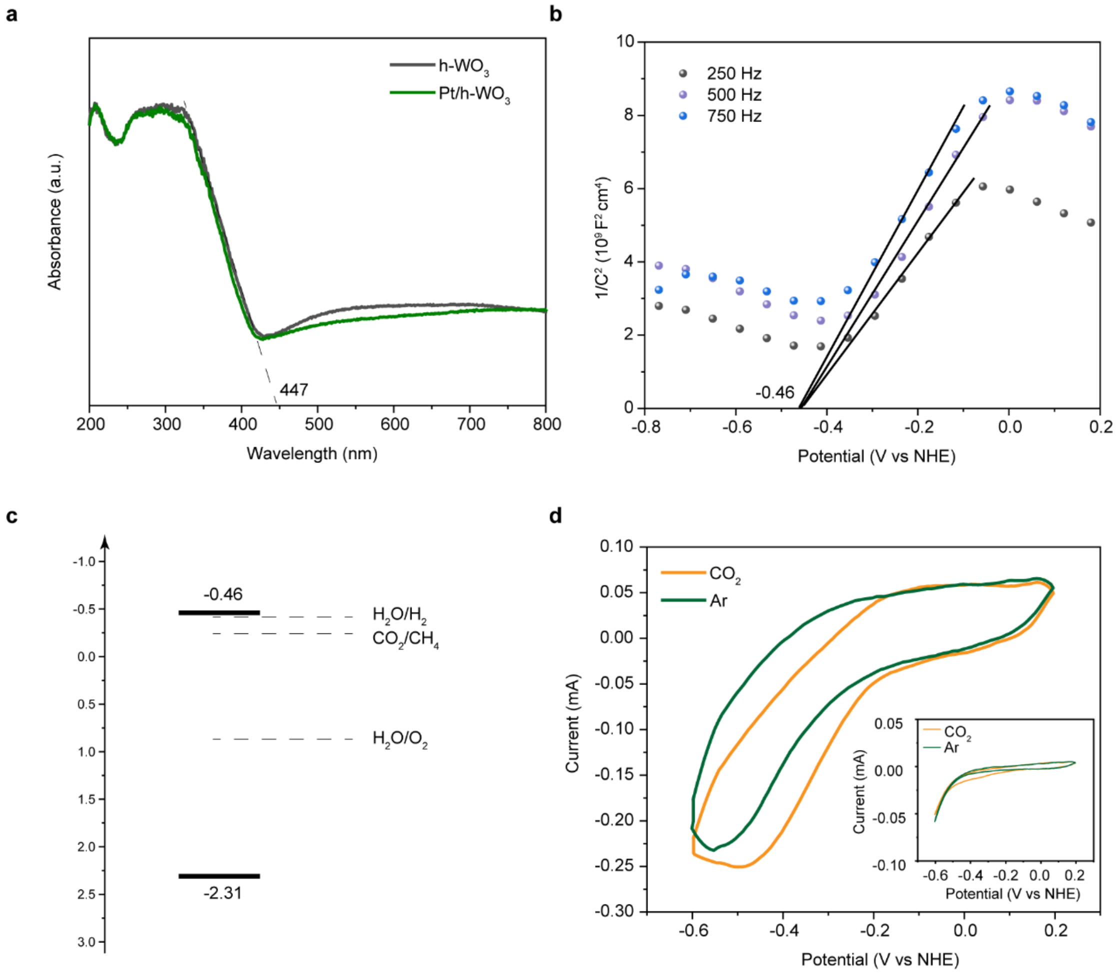

Supplementary Figure 9. (a) UV-visible diffuse reflectance spectroscopy of h-WO_3 and Pt/h-WO_3. (b) M–S plots and (c) band structure of Pt/h-WO_3. (d) CV curves of Pt/h-WO_3 in Ar-saturated or CO_2-saturated 0.1 M H_2SO_4 solution at 100 mV/s. The inserted figure is the result of a polished glassy carbon electrode without samples.

The light absorption of the obtained catalysts was analyzed by UV–vis diffuse reflectance spectroscopy. The band gap of Pt/h-WO_3 was determined to be 2.77 eV through the absorption edge using the Kubelka–Munk method. According to previous reports, tungsten trioxide is an n-type semiconductor, which means that the flat band potential lies very close to the bottom of the conduction band [4,5]. Therefore, the Mott–Schottky (M–S) plots of Pt/h-WO_3 were used to investigate the conduction band position of Pt/h-WO_3, and the result shows that the flat band potential of Pt/h-WO_3 is -0.46 V (vs. NHE, pH = 7). The M–S plots and band gap results show that the energy band location of Pt/h-WO_3 can meet the needs of water decomposition (-0.41 V vs. NHE, pH = 7) to produce hydrogen atoms for storage and the conversion of CO_2 to

CH_4 (-0.24 vs. NHE, pH = 7) [4]. Furthermore, we studied the generation and consumption of Pt/H_xWO_3 intermediates by CV curves. In the Ar-saturated electrolyte solution, the hydrogen atoms can effectively be inserted into the structure of Pt/h-WO_3, and this process is accompanied by the generation of Pt/H_xWO_3 products [6, 7]. Once the electrolyte solution is saturated with CO_2, the cathode current clearly increases. This indicates that CO_2 can be reduced at the nearly identical potential for the reduction of Pt/H_xWO_3. In addition, the cathode current on the bare glassy carbon electrode is much lower in both the Ar- or CO_2-saturated electrolyte solution. The above results indicate that the reduction capacity of Pt/H_xWO_3 also meets the needs of CO_2 conversion.

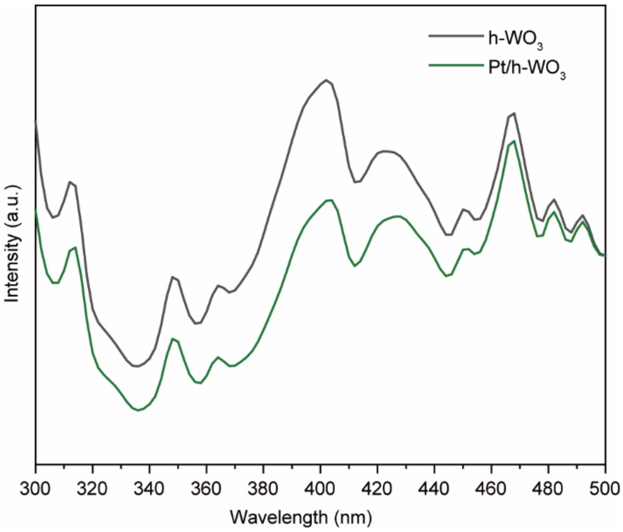

Supplementary Figure 10. Steady-state photoluminescence (PL) spectroscopy of h-WO_3 and Pt/h-WO_3 with an excitation wavelength of 280 nm.

The charge recombination of the Pt/h-WO_3 was suppressed after Pt was introduced, suggesting that the separation of photogenerated electrons and holes is improved.

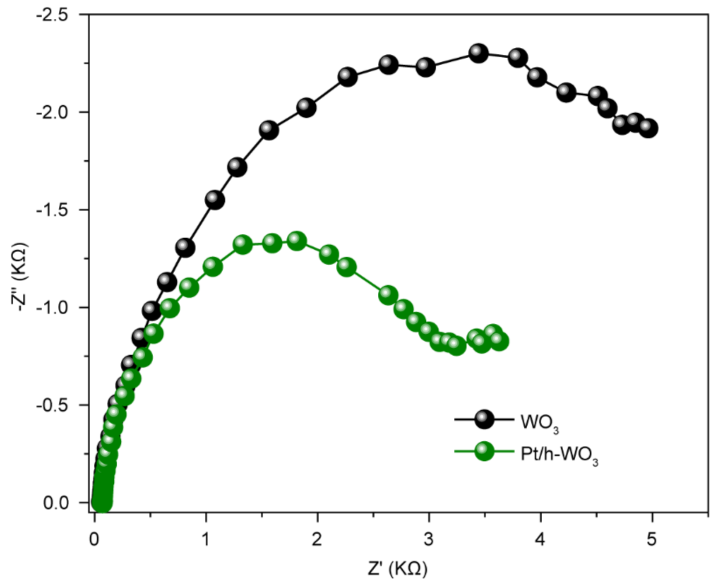

Supplementary Figure 11. Electrochemical impedance spectroscopy (EIS) Nyquist plots of h-WO_3 and Pt/h-WO_3 under dark conditions.

The arc radius of Pt/h-WO_3 on the EIS Nyquist plot is smaller than that of h-WO_3. This indicates that Pt/h-WO_3 has substantially smaller charge transfer resistance as compared to h-WO_3, mainly because Pt accelerates the transfer of electrons [8].

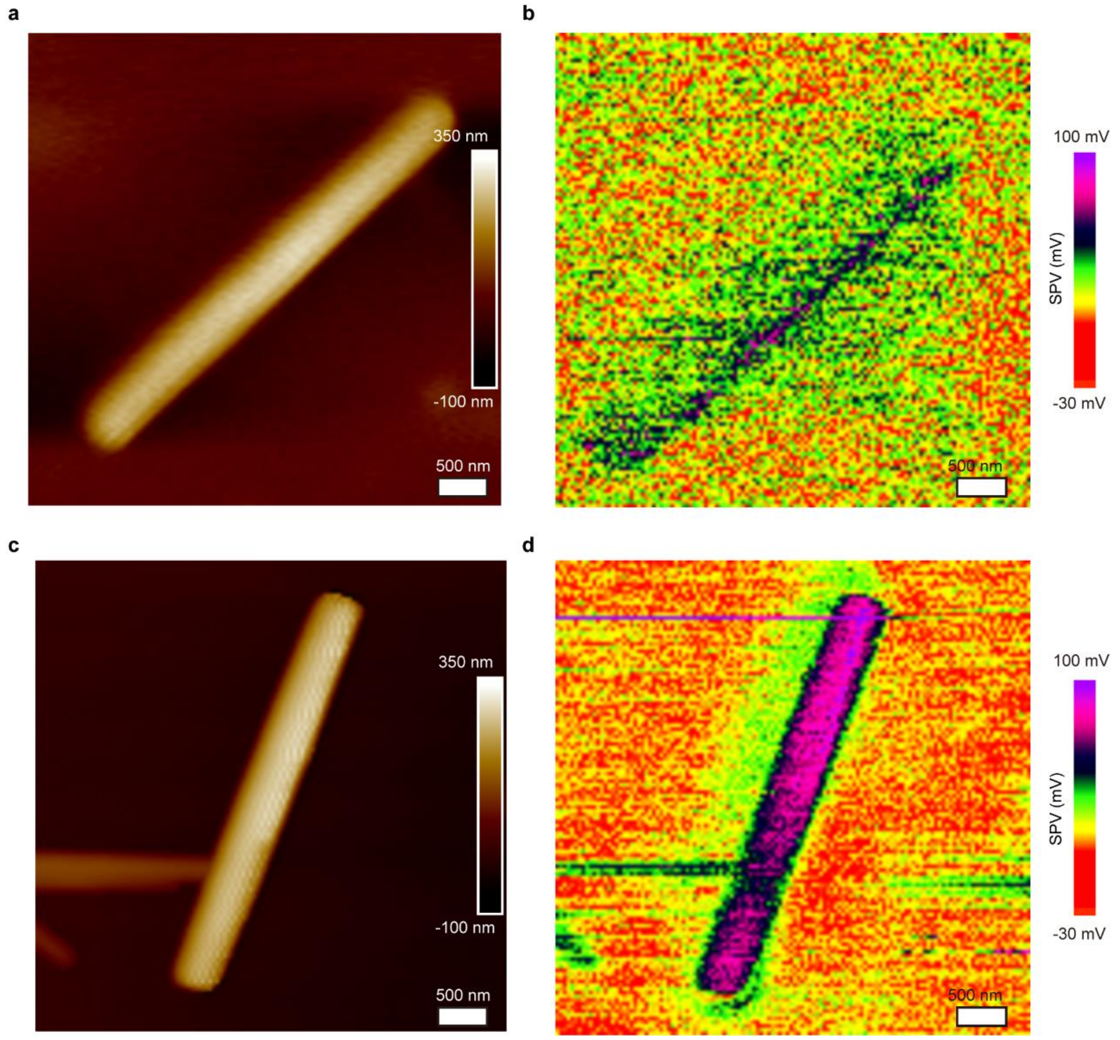

Supplementary Figure 12. AFM topography images of (a) h-WO_3 and (c) Pt/h-WO_3. SPV images of (b) h-WO_3 and (d) Pt/h-WO_3.

Both h-WO_3 and Pt/h-WO_3 show the SPV response under light illumination. The SPV images of h-WO_3 and Pt/h-WO_3 show the same features as the topography image. The SPVs of Pt/h-WO_3 and h-WO_3 are about 60 mV and 10 mV, respectively, indicating that more photogenerated electrons are localized on the surface of Pt/h-WO_3 [9].

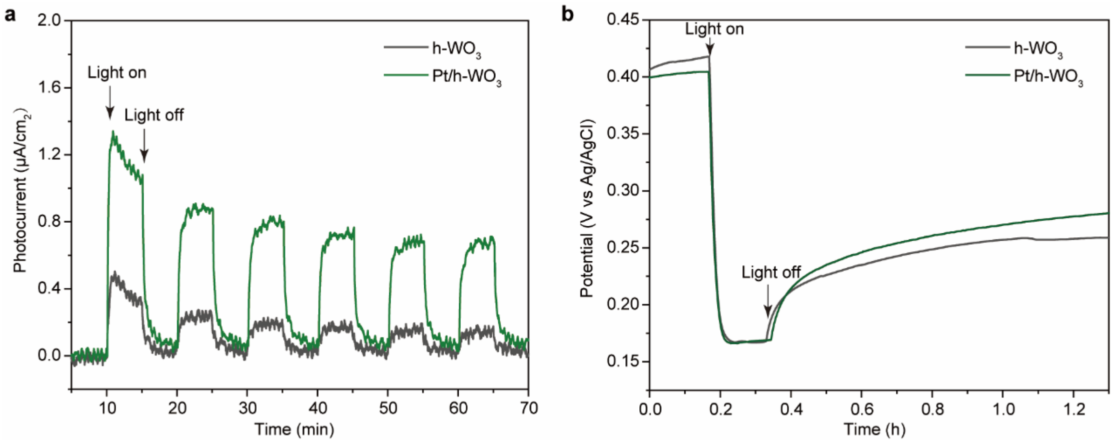

Supplementary Figure 13. (a) Transient photocurrent responses of Pt/h-WO_3. (b) Changes in the potential of h-WO_3 and Pt/h-WO_3 under a 10 min illumination followed by that in dark.

The photocurrents and potentials of the samples can be kept for a period of time after the end of illumination, indicating that the stored photogenerated electrons could be released spontaneously under dark condition.

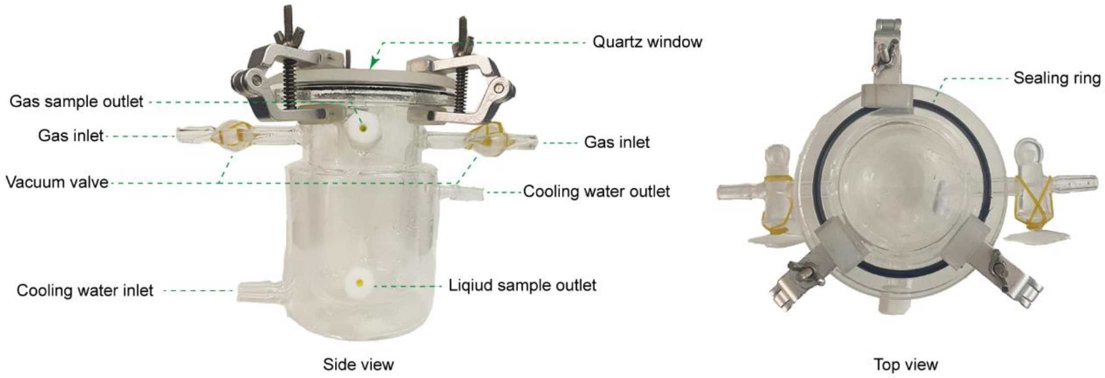

Supplementary Figure 14. Detailed configuration of reactor.

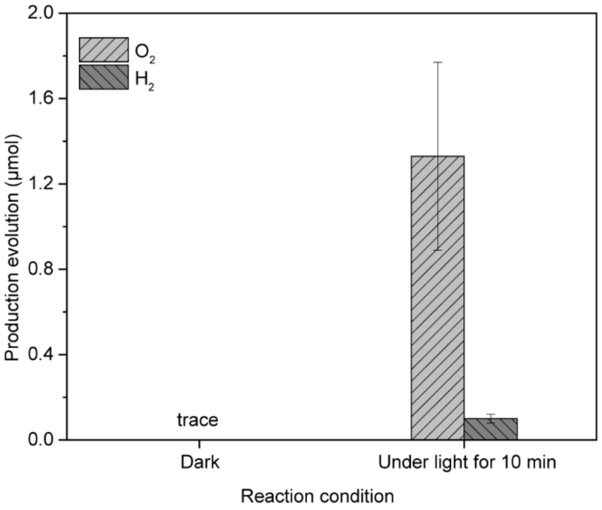

Supplementary Figure 15. O_2 and H_2 evolution on Pt/h-WO_3 in dark and under light illumination for 10 min. Error bars denote the standard deviation of data from three tests.

Note that in the process of oxygen detection, the reactor was filled with high-purity Ar as carrier gas for detection.

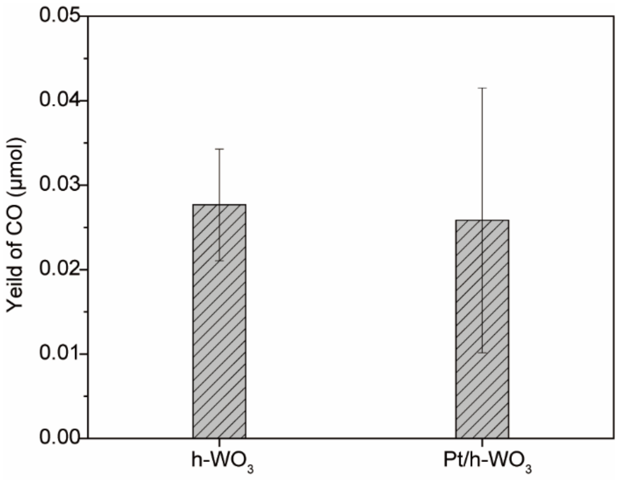

Supplementary Figure 16. CO evolution on Pt/h-WO_3 and h-WO_3 after 10 days of dark reaction. Error bars denote the standard deviation of data from three tests.

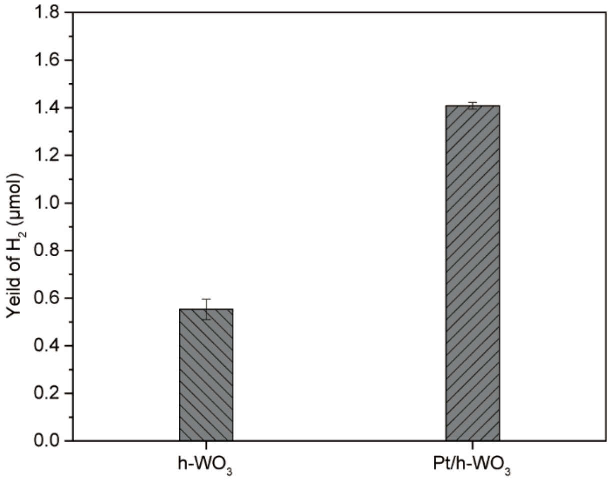

Supplementary Figure 17. H_2 evolution on Pt/h-WO_3 and h-WO_3 after 10 days of dark reaction. Error bars denote the standard deviation of data from three tests.

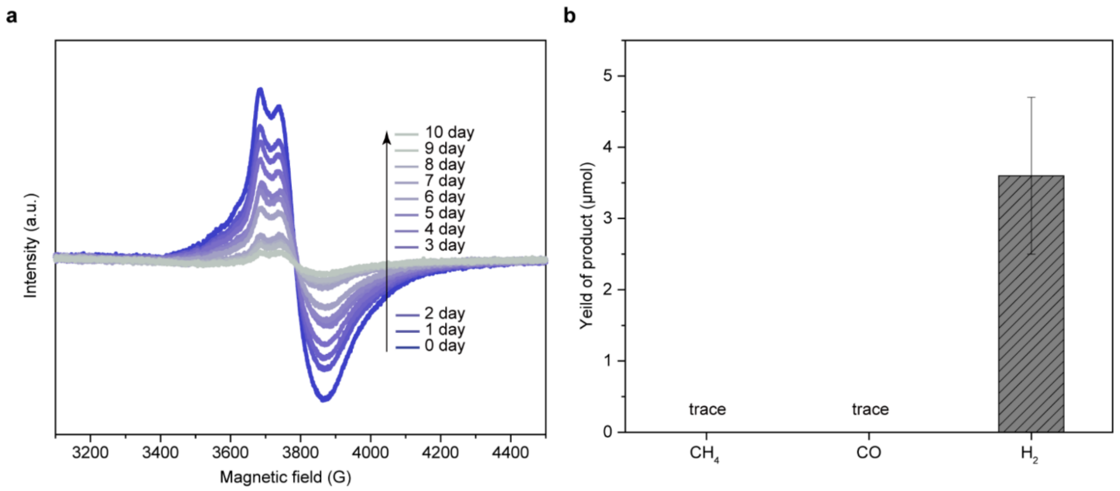

Supplementary Figure 18. (a) EPR spectra of Pt/h-WO_3 in the dark. (b) Products evolution on Pt/h-WO_3. The test conditions were the same as those in the CO_2 atmosphere except that high-purity CO_2 was replaced with high-purity Ar.

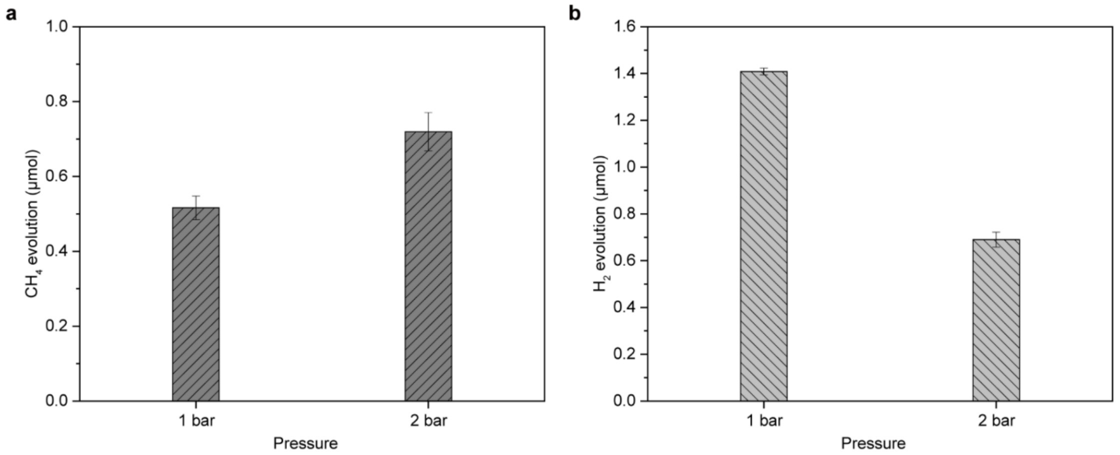

Supplementary Figure 19. (a) Yield of CH_4 and (b) H_2 over 10 mg Pt/h-WO_3 catalyst after 10 min of simulated sunlight irradiation at 1 bar and 2 bar absolute pressure. Error bars denote the standard deviation of data from three tests.

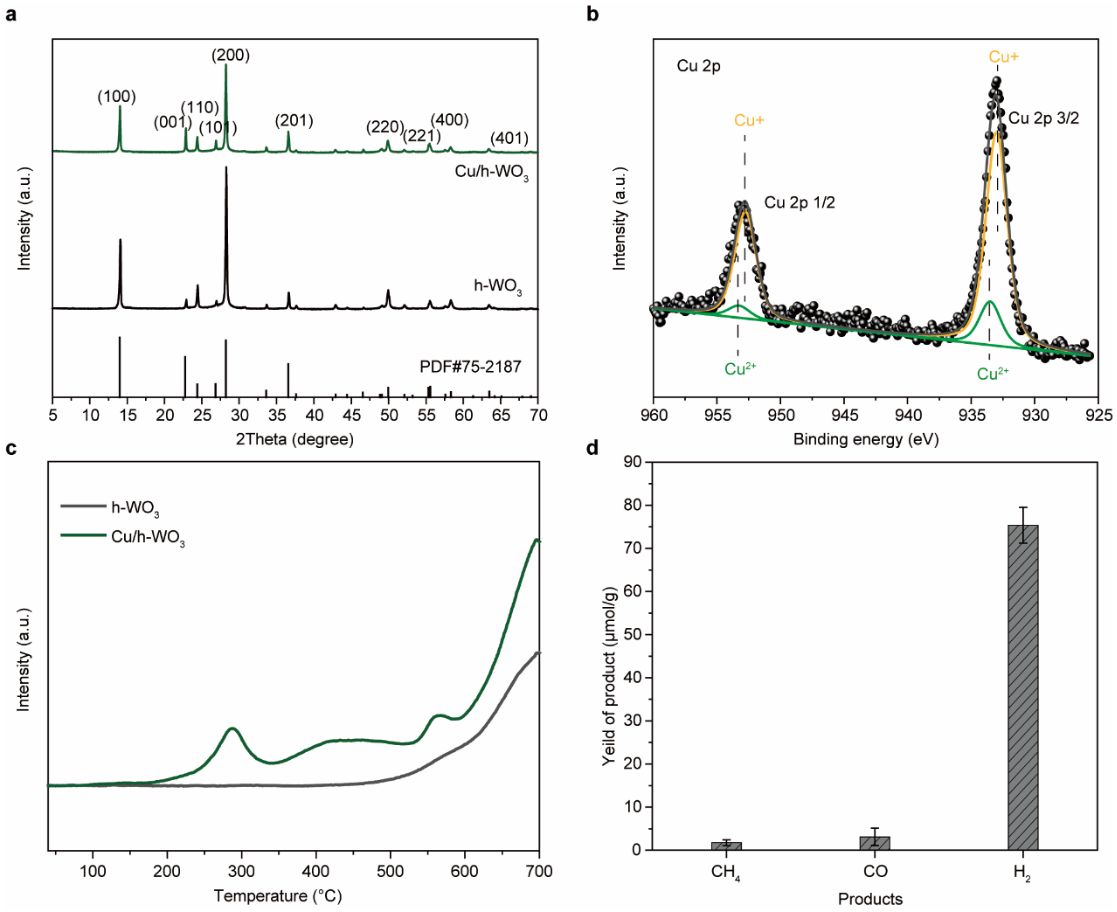

Supplementary Figure 20. (a) XRD patterns of Cu/h-WO_3 and h-WO_3. (b) High-resolution Cu 2p XPS spectra of Cu/h-WO_3. (c) H_2-TPR spectra of h-WO_3 and Cu/h-WO_3. (d) Products evolution on Cu/h-WO_3 in dark reaction.

The XRD peaks of Cu/h-WO_3 are the same as those of h-WO_3 while no other peaks appeared, indicating that the introduction of Cu atoms does not affect the structure of the h-WO_3 carrier. The Cu 2p XPS spectrum demonstrates the successful loading of Cu on the catalyst surface. From the H_2-TPR results, it can be seen that the introduction of Cu has the effect of hydrogen spillover similar to that of Pt. In addition, after the addition of Cu, the main product changed to H_2.

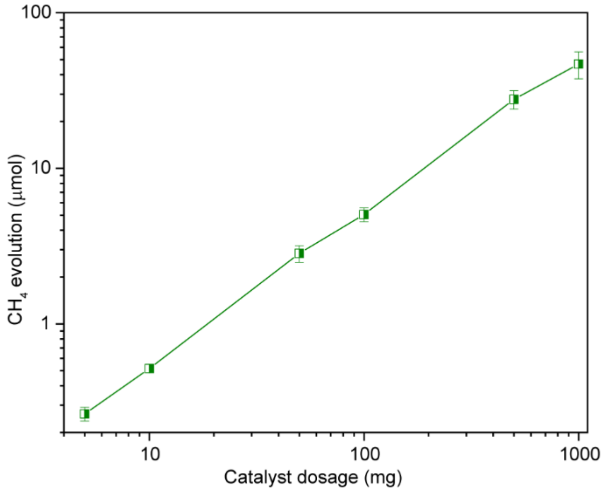

Supplementary Figure 21. Yield of CH_4 over catalyst with different dosage after dark reaction. Note that the illumination time should be extended appropriately to ensure that the H atoms stored in the catalyst are saturated. Error bars denote the standard deviation of data from three tests.

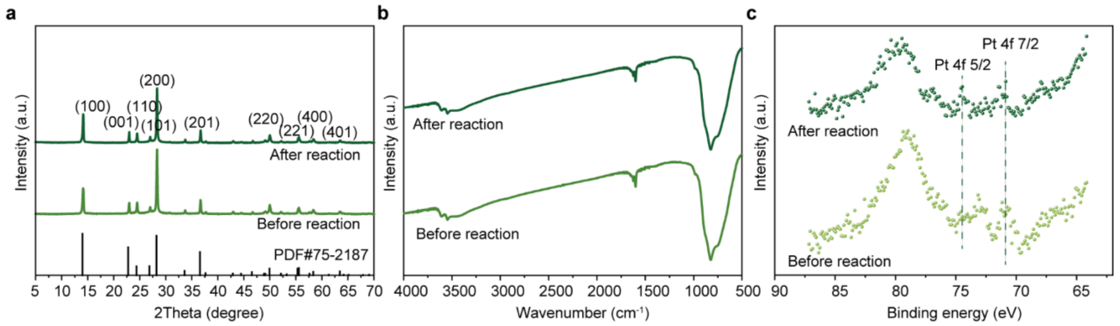

Supplementary Figure 22. (a) XRD patterns, (b) FTIR spectra, and (c) high-resolution Pt 4f XPS spectra of Pt/h-WO_3 before and after four cycle tests.

The XRD patterns, FT-IR spectra, and high-resolution Pt 4f XPS spectra of Pt/h-WO_3 did not change significantly during the photocatalytic reduction of CO_2, indicating that the catalyst has high recyclability.

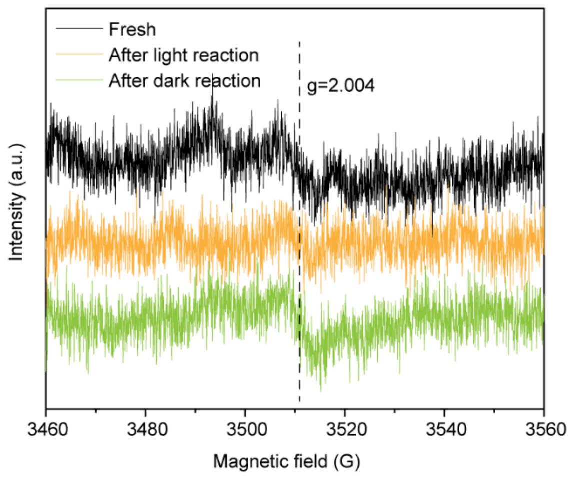

Supplementary Figure 23. EPR spectra of Pt/h-WO_3 before reaction, after light reaction and after dark reaction.

The sign of g factor around 2.004 is attributed to the oxygen vacancies [10]. According to the results of EPR test results, the concentration of oxygen vacancies in Pt/h-WO_3 sample did not change significantly during the reaction, indicating that no structural defects were created.

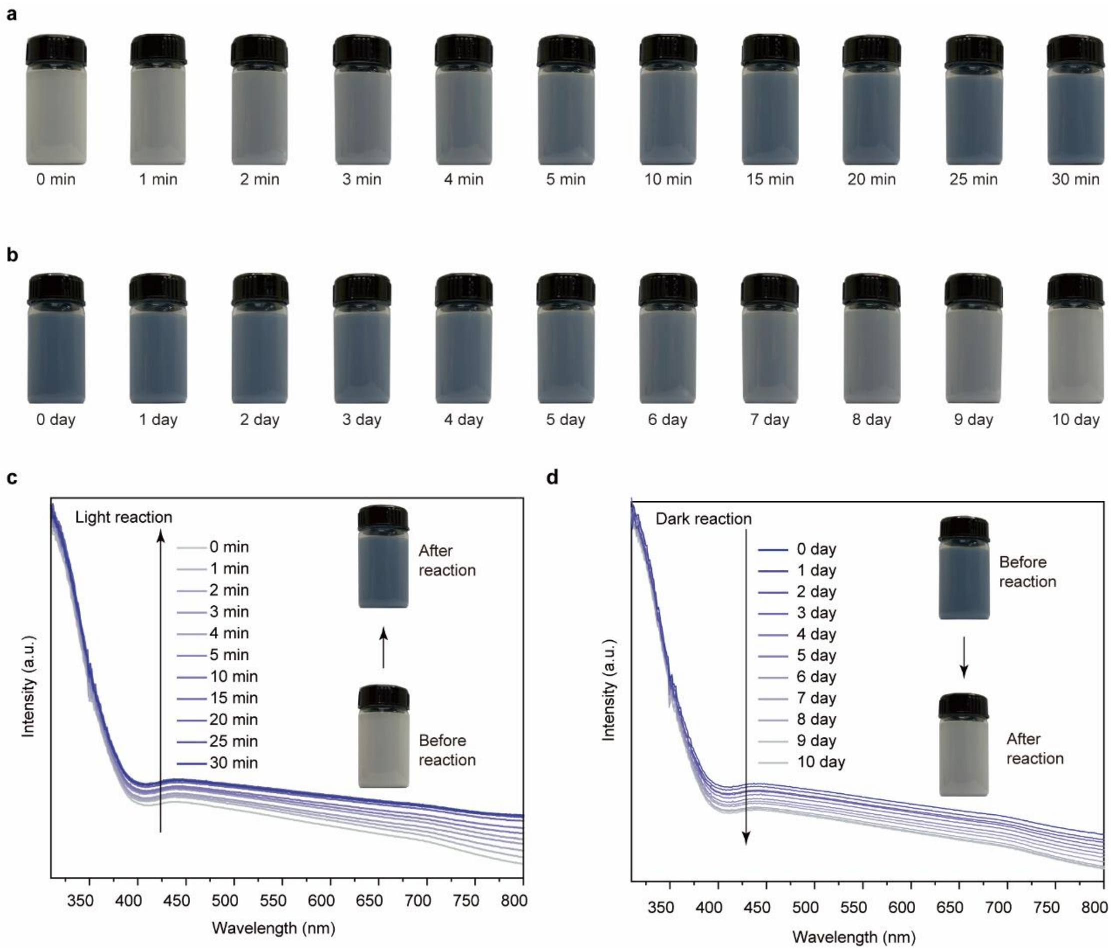

Supplementary Figure 24. The photographs of solution containing the Pt/h-WO_3 catalyst during(a) light reaction and (b) dark reactions. The change of UV-vis spectra for Pt/h-WO_3 upon (c) light irradiation and (d) dark condition.

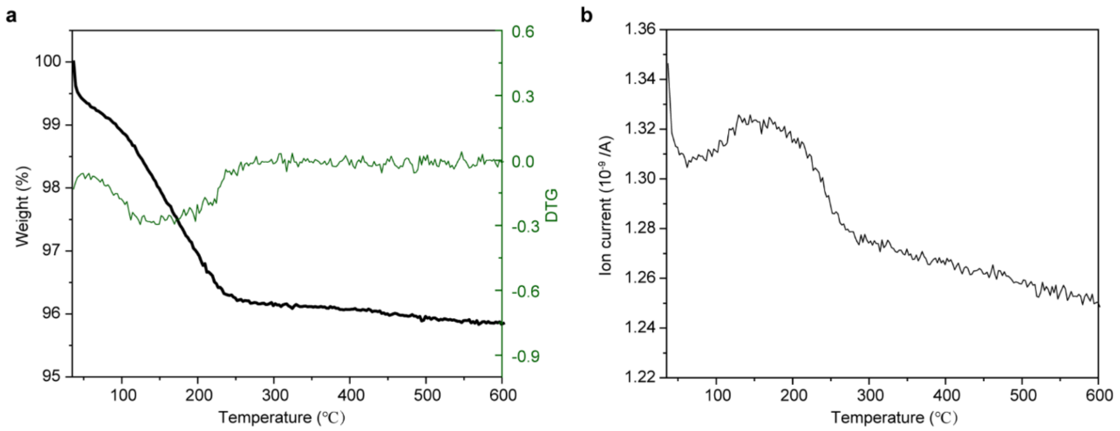

Supplementary Figure 25. (a) Thermogravimetric analysis results, and (b) H_2O (m/z = 18) release of Pt/h-WO_3.

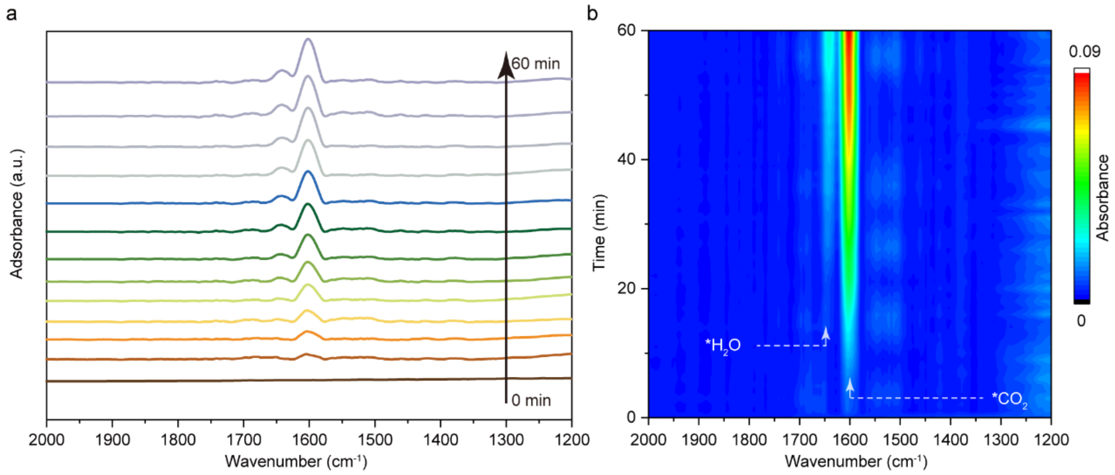

Supplementary Figure 26. (a) In situ FTIR spectra for the CO_2 reduction process in dark over Pt/h-WO_3 and (b) its corresponding 2D contour color fill.

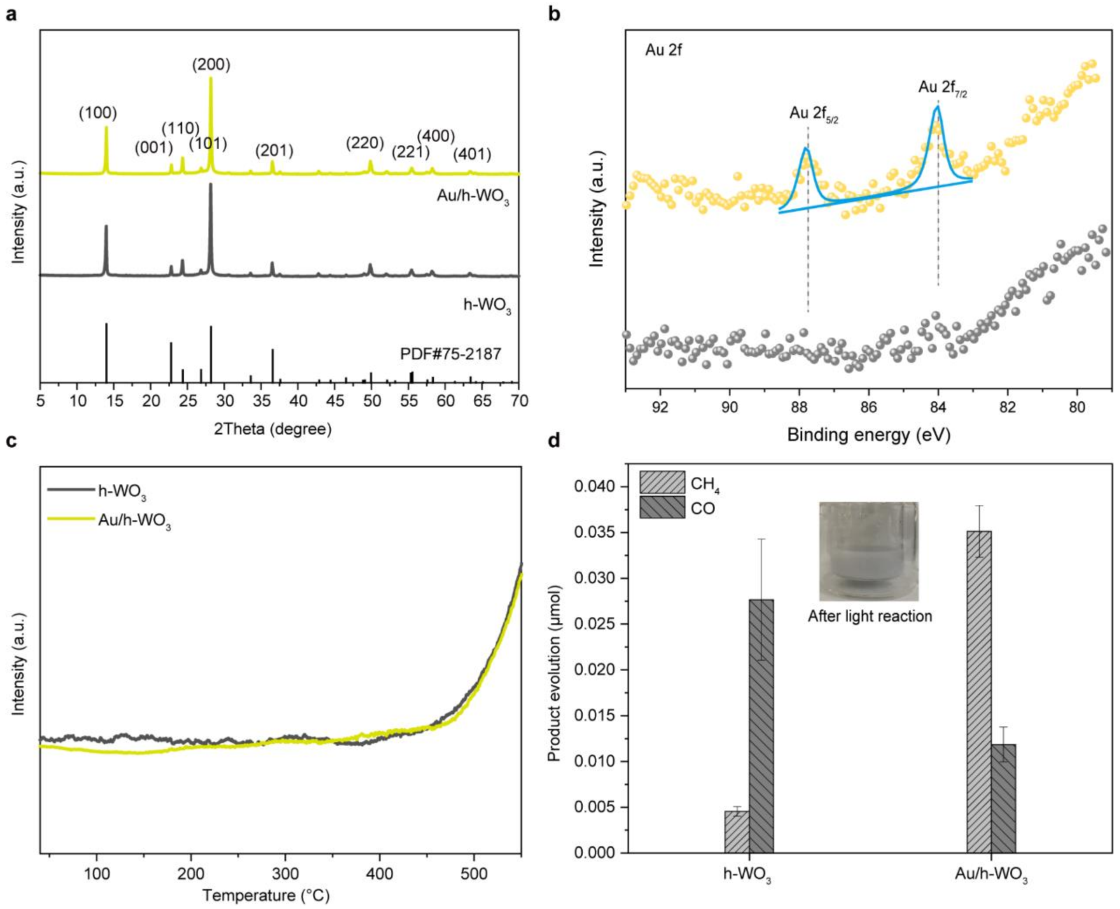

Supplementary Figure 27. Characterization of Au/h-WO_3. (a) XRD patterns of h-WO_3 and Au/h-WO_3. (b) Au 4f XPS spectra of h-WO_3 and Au/h-WO_3. (c) H_2-TPR spectra of h-WO_3 and Au/h-WO_3. (d) CO and CH_4 evolution from catalytic CO_2 reduction on h-WO_3 and Au/h-WO_3 in dark after 10 min of simulated sunlight irradiation. The inset figure shows the photograph of solution containing the Au/h-WO_3 catalyst after light reaction. Error bars denote the standard deviation of data from three tests.

The XRD pattern of Au/h-WO_3 shows that the addition of Au does not affect the structure of the h-WO_3 carrier. The Au 2f XPS spectrum of Au/h-WO_3 demonstrates the successful loading of Au on the catalyst surface. However, there is no reduction peak in H_2-TPR spectrum, which indicates that Au/h-WO_3 may not have the ability of hydrogen spillover to WO_3 carrier. After 10 min of illumination, the color of the solution containing Au/h-WO_3 catalyst was not changed obviously, indicating that the electrons and H atoms had not been stored efficiently, which is consistent with the H_2-TPR results. This conclusion was further supported by the fact that there was no distinct production of CO and CH_4 in dark.

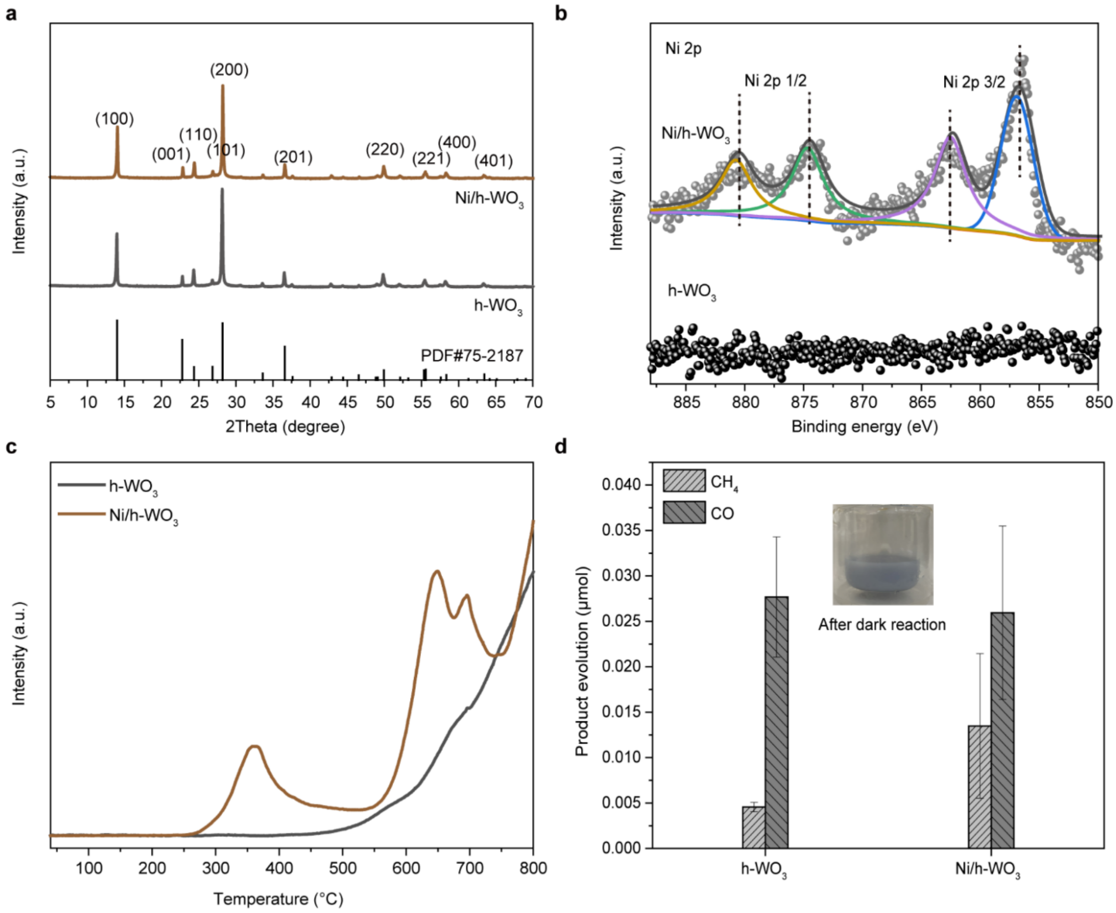

Supplementary Figure 28. Characterization of Ni/h-WO_3. (a) XRD patterns of h-WO_3 and Ni/h-WO_3. (b) Ni 2p XPS spectra of h-WO_3 and Ni/h-WO_3. (c) H_2-TPR spectra of h-WO_3 and Ni/h-WO_3. (d) CO and CH_4 evolution on Ni/h-WO_3 and h-WO_3 under dark condition for 10 days after 10 min of simulated sunlight irradiation. The inset figure shows the photograph of solution containing the Ni/h-WO_3 catalyst after dark reaction. Error bars denote the standard deviation of data from three tests.

The XRD pattern of Ni/h-WO_3 shows that the addition of Ni does not affect the structure of the h-WO_3 carrier. The deconvoluted Ni 2p XPS spectrum indicates the existence of metallic Ni and Ni^{2+} [11]. It can be seen from H_2-TPR spectrum that the Ni/h-WO_3 has the property of hydrogen spillover. However, the CO_2 conversion performance of Ni/h-WO_3 is nearly equal to that of bare h-WO_3, suggesting that the stored electrons and H atoms cannot be spontaneously released. After the reaction, the color of the solution is still pale blue, which proves that W^{5+} cannot be turned back to W^{6+}.

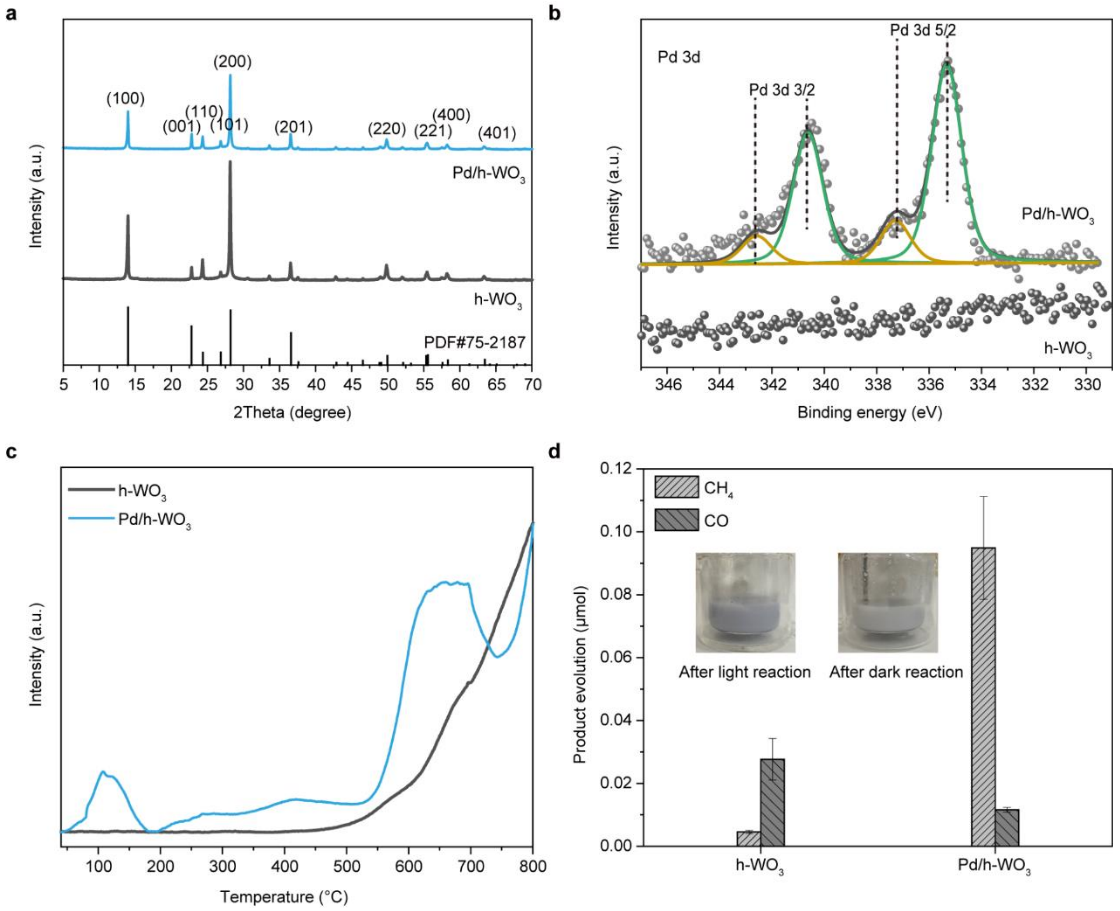

Supplementary Figure 29. Characterization of Pd/h-WO_3. (a) XRD patterns of h-WO_3 and Pd/h-WO_3. (b) Pd 3d XPS spectra of h-WO_3 and Pd/h-WO_3. (c) H_2-TPR spectra of h-WO_3 and Pd/h-WO_3. (d) CO and CH_4 evolution on Pd/h-WO_3 and h-WO_3 under dark condition after 10 min of simulated sunlight irradiation. The inset figure shows the photograph of solution containing the Pd/h-WO_3 catalyst after light reaction and dark reaction. Error bars denote the standard deviation of data from three tests.

The XRD peaks of Pd/h-WO_3 are the same as those of h-WO_3 while no other peaks appeared, indicating that the addition of Pd does not affect the structure of the h-WO_3 carrier. The Pd 3d XPS peaks of Pd/h-WO_3 are mainly located at 335.3 eV (Pd $3d_{3/2}$) and 340.4 eV (Pd $3d_{5/2}$), demonstrating the successful loading of Pd on the catalyst surface. From the H_2-TPR results, three new H_2 desorption peaks appeared for Pd/h-WO_3, suggesting the hydrogen spillover from Pd sites to WO_3 carrier. This phenomenon is consistent with that of Pt/h-WO_3, indicating that Pd can also induce electrons and H atoms storage. After loading Pd, the generation of CH_4 under dark conditions increased significantly, which indicates that the stored electrons and H atoms in Pd/h-WO_3 can be spontaneously released to achieve CO_2 reduction. This

process can also be demonstrated by the color changes of the solution containing the Pd/h-WO_3 catalyst.

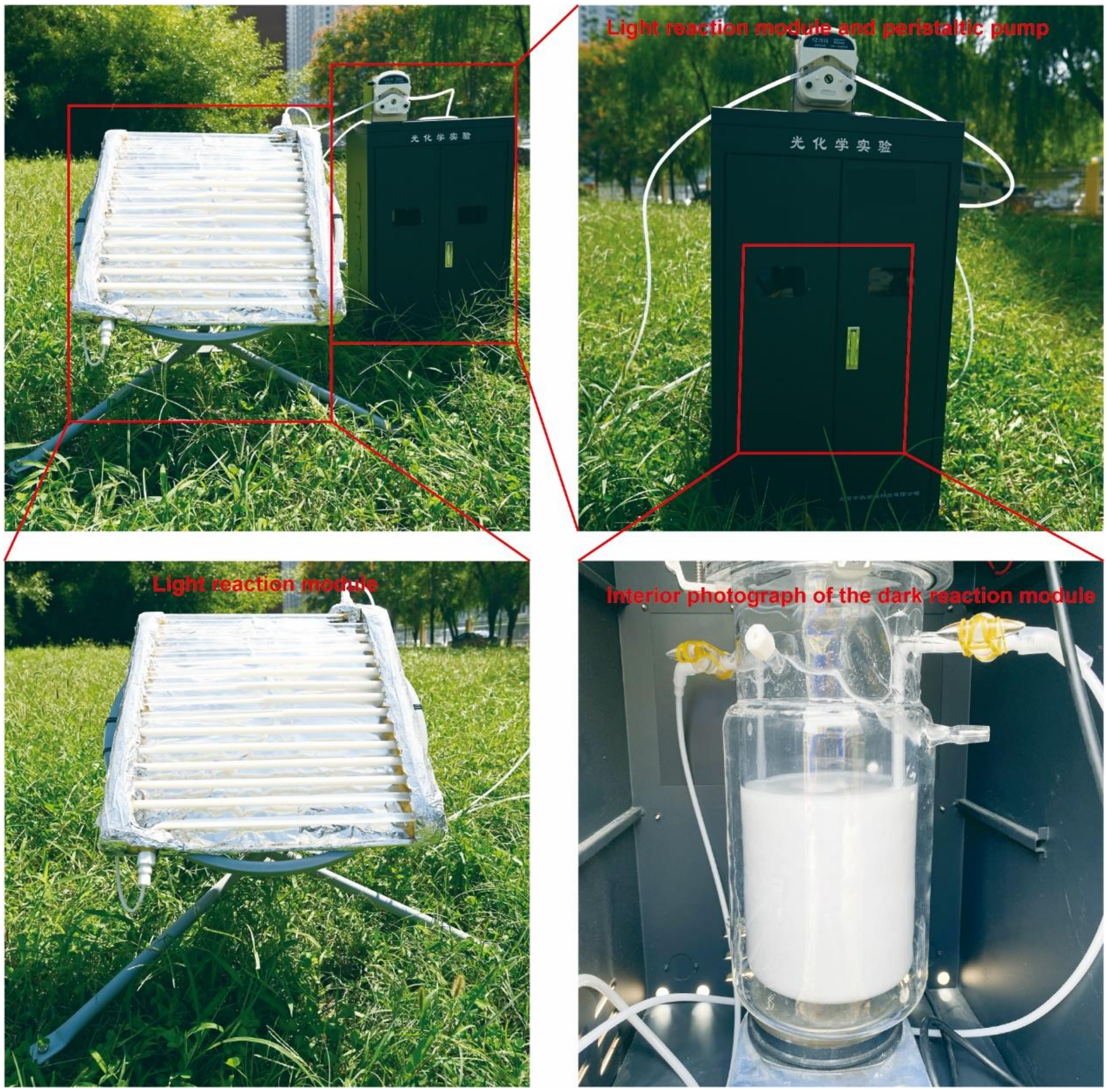

Supplementary Figure 30. The photographs of outdoor experimental equipment.

The optical absorption part consists of 15 quartz tubes (50 cm length and 1 cm inner diameter) connected alternately at the end. The quartz tubes were fixed on an iron stand. The light reaction module has no sun-following device and is fixed in one direction. The reactor for the dark reaction is a 6 L glass container with an off-line test sampling port at the top. For the demonstration experiment, 1 g of the Pt/h-WO_3 was dispersed in 3 L deionized water and sonicated for 30 min. Then the obtained solution transferred to the dark reaction module, which was then connected to the light reaction module by Teflon tube. Subsequently, the whole system was purged with high-purity CO_2 for 6 h to fully exclude air. During the test, 3 mL gas in the dark reaction model was extracted every morning and evening for detection.

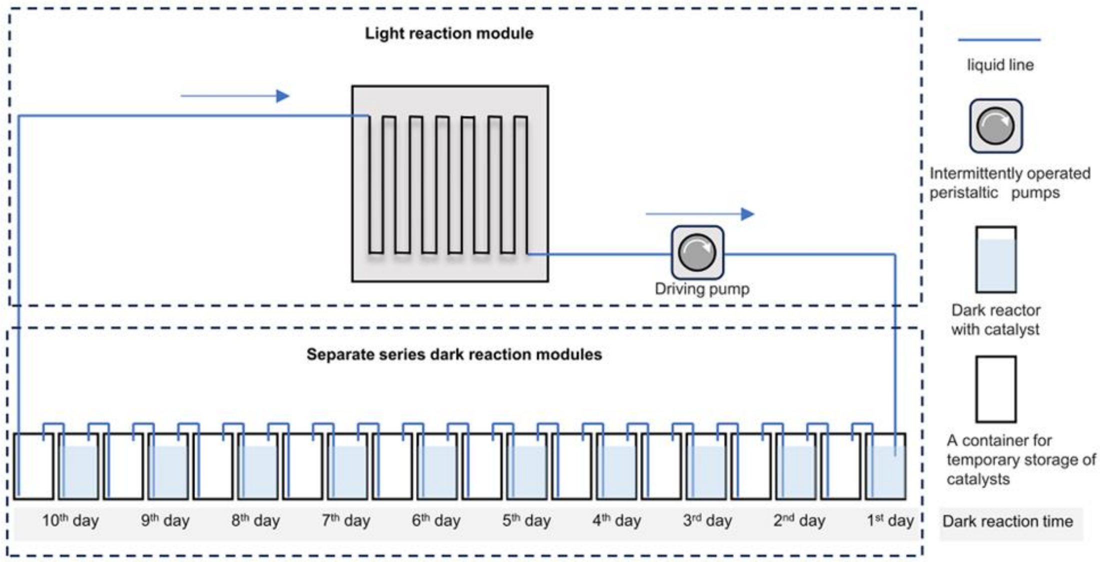

Supplementary Figure 31. Schematic diagram of improved outdoor experimental equipment.

There is still plenty of room for improvement in the usage scenario by taking advantage of the catalyst and avoiding its shortcomings. Here, we provide some ideas to improve the reaction device for reference in future research. Because the rate of light reaction is much faster than that of dark reaction, the stored hydrogen atoms and electrons quickly reach saturation and cannot be consumed in time. Therefore, we can increase the size of dark reaction module and increase the amount of catalyst undergoing dark reaction to improve the catalytic performance of the whole reaction system. In addition, it is a way to enhance catalytic efficiency by ensuring that the catalyst, which stores electrons and hydrogen atoms, is fully depleted before exposed to light for the light reaction process. Based on the above ideas, we can redesign the reaction module for the reference of subsequent research. First, the storage process of the catalyst is related to the light time, and increasing the amount of catalyst does not affect its storage capacity but requires a longer light irradiation time. Therefore, we can adjust the concentration of the catalyst and the area exposed to light to ensure that the catalyst in the light reaction module reaches a state of hydrogen and electron saturation under one day of irradiation. Next, by activating the peristaltic pump, the catalysts in the dark reactor are transferred to the next day's reactor, while the saturated catalyst is transported to the first day's dark reactor. Simultaneously, the

catalyst from the tenth reactor reenters the light reaction module for hydrogen atoms and electrons storage. In addition, there is a separate container between each reactor for transferring the catalysts to avoid mixing catalysts of different reaction days. This design effectively solves the problem that the catalyst becomes saturated quickly and wastes light energy, and maximizes the advantages of the catalyst.

Table S1 Comparison of the state-of-the-art works on photocatalytic CO_2 reduction into CH_4.

Catalyst	Reaction condition	CH_4 evolution	Reference
Pt/h-WO_3	10 min of irradiation by 300 W Xe lamp followed by 10 days in the dark	51.6 μmol g^{-1}	This work
0.5 %Pt-WO_3	Under continuous irradiation by 500 W Xe-lamp for 5 h	3.64 μmol/g	[12]
Pt@Def-CN	Under continuous irradiation by 500 W Xe-lamp	6.3 μmol g^{-1} h^{-1}	[8]
1.8PHTSO	Under continuous irradiation by a 300 W Xe-lamp coupled with an AM 1.5 filter	9.7 μmol g^{-1} h^{-1}	[13]
Pd-HPP-TiO_2	Under continuous irradiation by a 300 W Xe- lamp	48.0 μmol g^{-1} h^{-1}	[14]
CoFe/N-GC-800	Under continuous irradiation by a 300 W Xe-lamp	10.87 μmol h^{-1} g^{-1}	[15]
N-C@Co	Under continuous irradiation by a 300 W Xe lamp for 23 h	155.7 μmol g^{-1}	[16]
Pd_{1+NPS}-C_3N_4	Under continuous irradiation by 250 mW cm^{-2} Xe-lamp	20.3 μmol g^{-1} h^{-1}	[17]
Au/TiO_2/$W_{18}O_{49}$	Under continuous irradiation by 300 W Xe lamp	35.55 μmol g^{-1} h^{-1}	[18]
PtCu-crCN	Under continuous irradiation by a 300 W Xe lamp	2.8 μmol g^{-1} h^{-1}	[19]
CCN-W	Under continuous irradiation by a 300 W Xe arc lamp	4.45 μmol g^{-1} h^{-1}	[20]
Cu@Cu_2O/N-GC-600	Under continuous irradiation by a 300 W Xe-lamp for 7 h	38.89 μmol g^{-1}	[21]
0.7Ni-5OB-CN	Under continuous irradiation by a 300W Xe lamp	8.7 μmol g^{-1} h^{-1}	[22]
Cu_2O@WO_3	Under continuous irradiation by 300 W Xe lamp with a cutoff filter of 420 nm	59.1 μmol g^{-1} h^{-1}	[23]
3% Mo-WO_3	Under continuous irradiation by 500 W Xe-lamp, atmospheric CO_2 concentration	5.3 μmol g^{-1} h^{-1}	[24]
WO_3/$CdIn_eS_4$	Under continuous irradiation by 300 W Xe-lamp with a cutoff filter of 420 nm	1.6 μmol g^{-1} h^{-1}	[25]
0.8FePc/P-WO_3	Under continuous irradiation by 300 W Xe-lamp	~0.4 μmol g^{-1} h^{-1}	[26]

Table S2 The calculated amount of H based on ion exchange using 10 mg catalyst.

Catalyst	Fresh	After light reaction	After dark reaction
Pt/h-WO_3	0.86 μmol	8.48 μmol	0.92 μmol
h-WO_3	0.99 μmol	2.21 μmol	1.50 μmol

Table S3 Weather conditions during the outdoor test.

Number of days	date	Weather
1	7:00 AM, September 8−7:00 AM, September 9	In dark
2	7:00 AM, September 9−7:00 AM, September 10	sunny
3	7:00 AM, September 10−7:00 AM, September 11	cloudy
4	7:00 AM, September 11−7:00 AM, September 12	sunny
5	7:00 AM, September 12−7:00 AM, September 13	sunny
6	7:00 AM, September 13−7:00 AM, September 14	sunny
7	7:00 AM, September 14−7:00 AM, September 15	sunny
8	7:00 AM, September 15−7:00 AM, September 16	sunny
9	7:00 AM, September 16−7:00 AM, September 17	sunny
10	7:00 AM, September 17−7:00 AM, September 18	rainy
11	7:00 AM, September 18−7:00 AM, September 19	rainy
12	7:00 AM, September 19−7:00 AM, September 20	rainy
13	7:00 AM, September 20−7:00 AM, September 21	rainy
14	7:00 AM, September 21−7:00 AM, September 22	cloudy
15	7:00 AM, September 22−7:00 AM, September 23	sunny
16	7:00 AM, September 23−7:00 AM, September 24	cloudy

Supplementary references

1. Wang S, Gao Y and Miao S *et al.* Positioning the water oxidation reaction sites in plasmonic photocatalysts. *J Am Chem Soc* 2017; **139**: 11771-8.
2. Ulmer U, Dingle T and Duchesne PN *et al.* Fundamentals and applications of photocatalytic CO_2 methanation. *Nat Commun* 2019; **10**: 3169.
3. Besnardiere J, Ma B and Torres-Pardo A *et al.* Structure and electrochromism of two-dimensional octahedral molecular sieve h'-WO_3. *Nat Commun* 2019; **10**: 327.
4. Sun SM, Watanabe M and Wu J *et al.* Ultrathin WO_3 center dot $0.33H_2O$ nanotubes for CO_2 photoreduction to acetate with high selectivity. *J Am Chem Soc* 2018; **140**: 6474-82.
5. Zheng HD, Ou JZ and Strano MS *et al.* Nanostructured tungsten oxide - properties, synthesis, and applications. *Adv Funct Mater* 2011; **21**: 2175-96.
6. Kim DJ, Pyun SI. Hydrogen transport through rf-magnetron sputtered amorphous WO_3 film with three kinds of hydrogen injection sites. *Solid State Ionics* 1997; **99**: 185-92.
7. Patra A, Auddy K and Ganguli D *et al.* Sol–gel electrochromic WO_3 coatings on glass. *Mater Lett* 2004; **58**: 1059-63.
8. Shi X, Huang Y and Bo Y *et al.* Highly selective photocatalytic CO_2 methanation with water vapor on single-atom platinum-decorated defective carbon nitride. *Angew Chem Int Ed* 2022; **61**: e202203063.
9. Gao W, Li S and He H *et al.* Vacancy-defect modulated pathway of photoreduction of CO_2 on single atomically thin $AgInP_2S_6$ sheets into olefiant gas. *Nat Commun* 2021; **12**: 4747.
10. Lei B, Cui W and Chen P *et al.* C–Doping Induced Oxygen-Vacancy in WO_3 Nanosheets for CO_2 Activation and Photoreduction. *ACS Catal* 2022; **12**: 9670-8.
11. Manh-Hiep V, Sakar M and Chinh-Chien N *et al.* Chemically bonded Ni cocatalyst onto the S doped g-C_3N_4 nanosheets and their synergistic enhancement in H_2 production under snlight irradiation. *ACS Sustain Chem Eng* 2018; **6**: 4194-203.
12. Wang HP, Zhang L and Zhou YY *et al.* Photocatalytic CO_2 reduction over

platinum modified hexagonal tungsten oxide: Effects of platinum on forward and back reactions. *Appl Catal B: Environ* 2020; **263**: 118331.

13. Dong C, Lian C and Hu S *et al.* Size-dependent activity and selectivity of carbon dioxide photocatalytic reduction over platinum nanoparticles. *Nat Commun* 2018; **9**: 1252.

14. Ma Y, Yi X and Wang S *et al.* Selective photocatalytic CO_2 reduction in aerobic environment by microporous Pd-porphyrin-based polymers coated hollow TiO_2. *Nat Commun* 2022; **13**: 1400.

15. He L, Zhang W and Lv F *et al.* CoFe alloy nanoparticles encapsulated in a 3D honeycomb-like N-doped graphitic carbon framework for photocatalytic CO_2 reduction. *J Mater Chem A* 2022.

16. Ma M, Chen J and Huang Z *et al.* Intermolecular hydrogen bond modulating the selective coupling of protons and CO_2 to CH_4 over nitrogen-doped carbon layers modified cobalt. *Chem Eng J* 2022; **444**: 136585.

17. Liu P, Huang Z and Gao X *et al.* Synergy between palladium single atoms and nanoparticles via hydrogen spillover for enhancing CO_2 photoreduction to CH_4. *Adv Mater* 2022; **34**: e2200057.

18. Liu Q, Zhao X and Song X *et al.* Pd nanosheet-decorated 2D/2D g-C_3N_4/WO_3.H_2O S-scheme photocatalyst for high selective photoreduction of CO_2 to CO. *Inorg Chem* 2022; **61**: 4171-83.

19. Cheng L, Zhang P and Wen Q *et al.* Copper and platinum dual-single-atoms supported on crystalline graphitic carbon nitride for enhanced photocatalytic CO_2 reduction. *Chinese J Catal* 2022; **43**: 451-60.

20. Liang Y, Wu X and Liu X *et al.* Recovering solar fuels from photocatalytic CO_2 reduction over W^{6+}-incorporated crystalline g-C_3N_4 nanorods by synergetic modulation of active centers. *Appl Catal B: Environ* 2022; **304**: 120978.

21. He L, Zhang W and Zhao K *et al.* Core–shell Cu@Cu_2O nanoparticles embedded in 3D honeycomb-like N-doped graphitic carbon for photocatalytic CO_2 reduction. *J Mater Chem A* 2022; **10**: 4758-69.

22. Wang Y, Qu Y and Qu B *et al.* Construction of six-oxygen-coordinated single Ni

sites on g-C_3N_4 with boron-oxo species for photocatalytic water-activation-induced CO_2 reduction. *Adv Mater* 2021; **33**: e2105482.

23. Hao JY, Qi BJ and Wei JJ *et al.* A Z-scheme Cu_2O/WO_3 heterojunction for production of renewable hydrocarbon fuel from carbon dioxide. *Fuel* 2021; **287**: 119439.

24. Wang HP, Zhang L and Wang KF *et al.* Enhanced photocatalytic CO_2 reduction to methane over $WO_3·0.33H_2O$ via Mo doping. *Appl Catal B: Environ* 2019; **243**: 771–9.

25. Zhang ZZ, Cao YX and Zhang FH *et al.* Tungsten oxide quantum dots deposited onto ultrathin $CdIn_2S_4$ nanosheets for efficient S-scheme photocatalytic CO_2 reduction via cascade charge transfer. *Chem Eng J* 2022; **428**: 131218.

26. Li B, Sun LQ and Bian J *et al.* Controlled synthesis of novel Z-scheme iron phthalocyanine/porous WO_3 nanocomposites as efficient photocatalysts for CO_2 reduction. *Appl Catal B: Environ* 2020; **270**: 118849.
